# Iridium Single Atoms to Nanoparticles: Nurturing the Local Synergy with Cobalt‐Oxide Supported Palladium Nanoparticles for Oxygen Reduction Reaction

**DOI:** 10.1002/advs.202404076

**Published:** 2024-06-27

**Authors:** Dinesh Bhalothia, Che Yan, Nozomu Hiraoka, Hirofumi Ishii, Yen‑Fa Liao, Sheng Dai, Po‐Chun Chen, Tsan‐Yao Chen

**Affiliations:** ^1^ Department of Engineering and System Science National Tsing Hua University Hsinchu 30013 Taiwan; ^2^ National Synchrotron Radiation Research Center Hsinchu 30076 Taiwan; ^3^ School of Chemistry & Molecular Engineering East China University of Science and Technology Shanghai 200237 P. R. China; ^4^ Department of Materials and Mineral Resources Engineering National Taipei University of Technology Taipei 10608 Taiwan

**Keywords:** fuel cells, in situ PFY XAS, mass activity, oxygen reduction reaction, single atoms

## Abstract

A ternary catalyst comprising Iridium (Ir) single‐atoms (SA)s decorated on the Co‐oxide supported palladium (Pd) nanoparticles (denoted as CPI‐SA) is developed in this work. The CPI‐SA with 1 wt.% of Ir exhibits unprecedented high mass activity (MA) of 7173 and 770 mA mg_Ir_^−1^, respectively, at 0.85 and 0.90 V versus RHE in alkaline ORR (0.1 m KOH), outperforming the commercial Johnson Matthey Pt catalyst (J.M.‐Pt/C; 20 wt.% Pt) by 107‐folds. More importantly, the high structural reliability of the Ir single‐atoms endows the CPI‐SA with outstanding durability, where it shows progressively increasing MA of 13 342 and 1372 mA mg_Ir_^−1^, respectively, at 0.85 and 0.90 V versus RHE up to 69 000 cycles (3 months) in the accelerated degradation test (ADT). Evidence from the in situ partial fluorescence yield X‐ray absorption spectroscopy (PFY‐XAS) and the electrochemical analysis indicate that the Ir single‐atoms and adjacent Pd domains synergistically promote the O_2_ splitting and subsequent desorption of hydroxide ions (OH^−^), respectively. Whereas the Co‐atoms underneath serve as electron injectors to boost the ORR activity of the Ir single‐atoms. Besides, a progressive and sharp drop in the ORR performance is observed when Ir‐clusters and Ir nanoparticles are decorated on the Co‐oxide‐supported Pd nanoparticles.

## Graphical abstract

The Ir single‐atoms and adjacent Pd domains synergistically promote the O_2_ splitting and subsequent desorption of hydroxide ions (OH^−^), respectively. Whereas, the Co‐atoms underneath serve as electron injectors to boost the oxygen reduction reaction activity of the Ir single‐atoms.

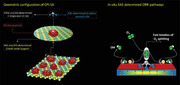

## 1 Introduction

Fuel cells have emerged as a promising clean energy conversion system, offering a potential solution to reduce reliance on fossil fuels and contribute to a sustainable energy future. Despite eye‐catching progress in recent decades, a critical challenge hindering the widespread market introduction of fuel cell technology is the lack of cost‐effective and durable electrocatalysts that can efficiently drive the sluggish oxygen reduction reaction (ORR) at the cathode.^[^ ^1^, ^2^ ^]^ Addressing this challenge, a novel approach involves the utilization of single‐atom catalysts (SACs), which have garnered attention as a highly promising category.^[^ ^3^, ^4^ ^]^ In contrast to the prevailing research focus on platinum (Pt)‐based catalysts, SACs offer a compelling alternative. The inherent advantages of SACs, including their efficient metal utilization and well‐defined active sites, make them a promising solution to overcome the limitations associated with traditional catalysts.^[^ ^5^, ^6^ ^]^ However, it is important to acknowledge that SACs come with certain limitations. One primary constraint is their relatively low metal content, which can affect their overall catalytic performance.^[^ ^7^ ^]^ Moreover, the isolated metal atoms within SACs can be more susceptible to various challenges, including aggregation, oxidation, and other structural changes.^[^ ^8^ ^]^ These issues can compromise the long‐term stability of the catalyst, potentially impacting its effectiveness over time. More importantly, the absence of ensemble sites represents a major drawback of SACs, especially in multistep reactions like ORR.^[^ ^9^ ^]^ To address the aforementioned issues, the synergistic potential of SACs in combination with other active species, such as other single atoms (SA)s, nanoclusters (NC)s, or nanoparticles (NP)s has opened up exciting opportunities for enhancing the efficiency and effectiveness of complex catalytic reactions.^[^ ^10^, ^11^, ^12^ ^]^ The synergistic interaction is defined as a phenomenon where the co‐existence of two or more distinct active species results in a pronounced catalytic performance.^[^ ^13^ ^]^ Such an enhanced catalytic activity is achieved via two different pathways. In the first case, the Sabatier principle becomes dominant, where, one of the active species can induce modifications in the electronic and geometric structures of the other species, ultimately leading to the optimized binding strength between reaction intermediates and the catalyst's surface, hence, an overall boost in the catalytic performance achieved.^[^ ^14^ ^]^ Besides, differing from the first case, both of the active sites can also directly participate in the reaction where different active sites are responsible for different reaction steps.^[^ ^15^ ^]^ Such a design enables the simultaneous operation of all intermediate steps, as a result, leading to a quantum leap in the catalytic performance of electrocatalysts. For instance, our previous works demonstrated the significantly enhanced ORR performance of Co@Pd core‐shell catalyst after the surface decoration of Pt‐trimers and Pt‐dimers.^[^ ^16^, ^17^ ^]^ Moreover, the potential synergism between nanoclusters (Pt and Ir) and Pd nanoparticles has been demonstrated previously.^[^ ^18^, ^19^, ^20^ ^]^ Furthermore, considering the technological importance of Iridium (Ir) and Nickel (Ni) in the electrocatalytic reaction, various compositions and configurations of Ir and Ni‐atoms along with dual metal dimers and metal clusters are also reported.^[^ ^21^, ^22^, ^23^, ^24^, ^25^ ^]^ However, while there have been sporadic reports on the successful integration of multiple components into a unified catalytic system, a systematic examination of the combination and synergy between single atoms, atomic clusters, and nanoparticles, backed by compelling in situ and operando evidence has been lacking.

Following this trail, Herein, we explore the key interactions of Ir‐SAs, Ir‐NCs, and Ir‐NPs in a ternary system by using the mesmerizing in situ partial fluorescence yield mode X‐ray absorption spectroscopy (PFY‐XAS) analysis. For the optimum scenario, the cobalt‐oxide‐supported Pd NPs with the surface decoration of Ir‐SAs (henceforth denoted as CPI‐SA) achieved the mass activity (MA) of as high as 7173 mAmg_Ir_^−1^ at 0.85 V versus RHE and 770 mAmg_Ir_^−1^ at 0.90 V versus RHE in 0.1 m KOH electrolyte toward ORR, which are respectively 107‐ and 31‐folds improved as compared to that of commercial J.M.‐Pt/C catalyst (67 and 24.9 mA mg^−1^). Of utmost importance, it exhibits unprecedented durability in an accelerated degradation test (ADT) and retains its 186% performance as that of pristine condition for 69k cycles. The cross‐referencing results of physical inspections, electrochemical characterizations, and in situ partial fluorescence yield mode X‐ray absorption spectroscopy (PFY‐XAS) analysis indicate that the high ORR performance of CPI‐SA catalyst originates from the potential synergy between the Ir‐SAs and Pd NPs, where Ir‐SAs boost the O_2_ splitting while Pd NPs promote the subsequent relocation of OH^−^ ions. Meanwhile, the Co‐oxide support supplies electrons for surface active sites. On the other hand, when the dimension of Ir‐species is increased, a significantly suppressed ORR performance is observed and can be attributed to the sluggish kinetics of O_2_ splitting on Ir‐NCs as compared to Ir‐SAs. Unsurprisingly, further increasing the dimension of Ir‐species to NPs results in surface oxidation of active sites, and therefore no ORR is observed for this case. In brief, the present work not only contributes to a highly efficient and economically competitive ORR catalyst but also provides the fundamental understanding of structure–performance relationship and thus offers advancements in scientific and industrial developments for ORR catalysis.

## 2 Results and Discussion

### 2.1 The Physical Structure Inspection

**Figure 1:**
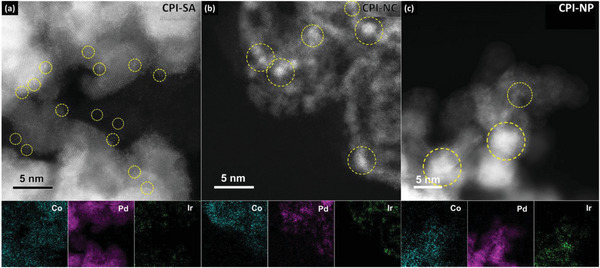
The high angle annular dark‐field scanning transmission electron microscopy HAADF‐STEM images and corresponding EDS elemental maps of Co, Pd, and Ir for the a) CPI‐SA, b) CPI‐NC, and c) CPI‐NP. Ir decorations are indicated by the yellow circles in the HAADF‐STEM images.

The morphology and structure information of the obtained CPI catalysts were carefully examined by aberration‐corrected scanning transmission electron microscopy (AC‐STEM). **Figure** **1**a shows the high angle annular dark‐field (HAADF)‐STEM and the corresponding energy‐dispersive X‐ray spectroscopy (EDS) elemental mapping result of the CPI‐SA, where the EDS elemental maps of Co and Pd demonstrate the formation of Pd NPs on Co‐oxide support. Notably, plenty of speckled bright dots (as indicated by the yellow circles in Figure 1a) on the Pd domains, exhibiting the high Z contrast were observed in the HAADF‐STEM image. These species could be determined as “Ir” due to the highest Z number (*Z* = 77) in the ternary system and are complimentary confirmed by the EDS map of “Ir” in Figure 1a, confirming the atomic isolation of Ir in the obtained CPI‐SA catalyst. For comparison, representative HAADF‐STEM images of CPI‐NC (3 wt.% Ir loading) and CPI‐NP (7 wt.% Ir loading) catalysts are provided in Figure 1b,c, respectively. In these figures, the decorated Ir species are denoted by the yellow circles and are confirmed by the corresponding EDS maps. Clearly, the size of decorated Ir‐species increases as the “Ir” loading goes up, where they formed nanoclusters (≈1.0–1.5 nm) in CPI‐NC and nanoparticles (≈3.0–5.0 nm) in CPI‐NP. These results are consistent with our experiment design and confirm that Ir decoration at different dimensions was successfully obtained in the CPI catalysts via the precise control in our synthesis approach. The STEM images of reference samples (Pd‐CNT and Co@Pd) are depicted in Figure S1 (Supporting Information). Furthermore, the crystal structures of the CPI catalysts are elucidated by the XRD analysis (Figure S2, Supporting Information), where changing the crystal structure of CPI catalysts with increasing Ir‐dosage confirms that the Ir‐species are decorated in different dimensions. The surface compositions of the CPI catalysts are revealed by the XPS (Figure S3, Tables S1 and S2, Supporting Information) analysis (Note S6, Supporting Information), where the composition of Ir within a probing depth of 1.5 nm in the CPI catalysts is 9.57% for CPI‐SA, 7.51% for CPI‐NC and 6.41% for CPI‐NP. Compared to that of Inductively coupled plasma‐atomic emission spectrometer (ICP‐AES) analysis (Table S3, Supporting Information), the substantially higher contents confirm that Ir atoms are decorated on the surface of CPI‐SA and CPI‐NC while a lower content suggests the intercalation of Ir atoms in the bulk of CPI‐NP.

**Figure 2:**
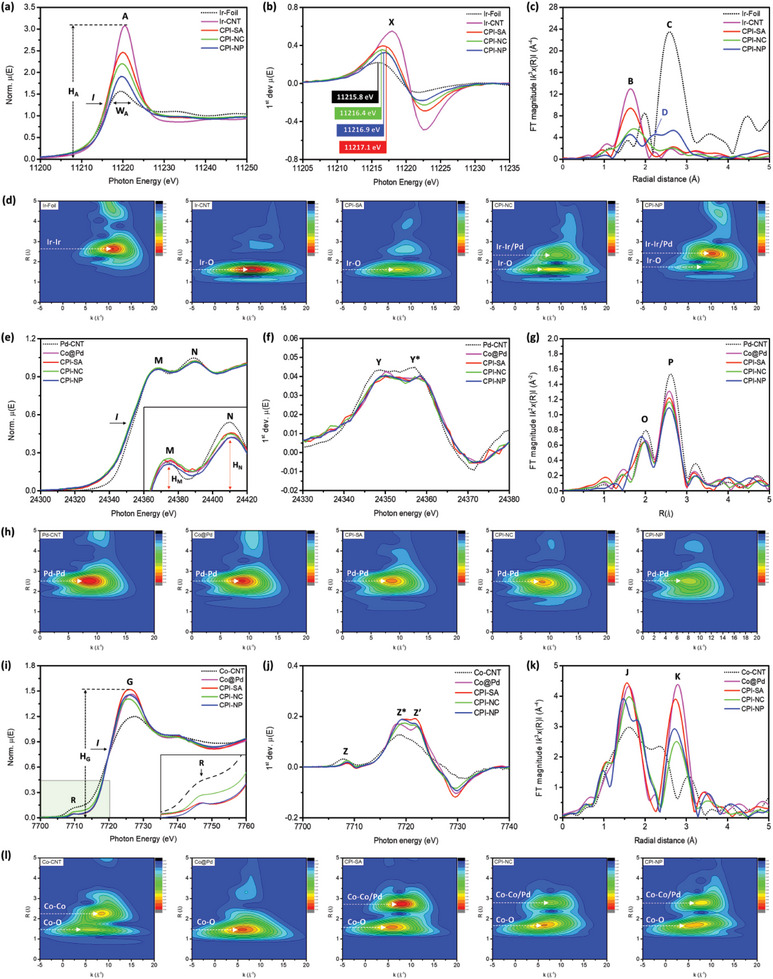
X‐ray absorption spectroscopy of CPI catalysts compared with reference samples. a) XANES, b) 1st derivative, c) FT‐EXAFS spectra, and d) WT patterns of CPI catalysts and reference samples (Ir‐Foil and Ir‐CNT) at Ir L_3_‐edge. e) XANES, f) 1st derivative, g) FT‐EXAFS spectra, and h) WT patterns of CPI catalysts and reference samples (Pd‐CNT and Co@Pd) at Pd K‐edge. i) XANES, j) 1st derivative, k) FT‐EXAFS spectra, and l) WT patterns of CPI catalysts and reference samples (Co‐CNT and Co@Pd) at Co K‐edge.

The X‐ray absorption spectroscopy (XAS) is carried out to shed further light on the local geometric configuration and electronic environment of Ir atoms in CPI catalysts. **Figure** **2**a depicts the normalized X‐ray absorption near‐edge spectra (XANES) of CPI catalysts at Ir L_3_‐edge, while the XANES spectra of Ir‐foil and Ir‐CNT are also compared for reference. Given that the white line intensity (H_A_) in a typical Ir L_3_‐edge spectrum corresponds to the unoccupied density of Ir‐d orbitals (i.e., with the increasing H_A_, the number of electrons in the occupied d band decreases) and the amount of surface chemisorption of oxygen.^[^ ^26^ ^]^ It is frequently reported in the literature that the high density of unoccupied d‐orbitals favors the electrocatalytic activity of catalysts.^[^ ^27^, ^28^ ^]^ Notably, the higher white line intensity of CPI‐SA suggests the highest density of unoccupied d‐orbitals and thus high ORR performance as compared to CPI‐NC and CPI‐NP catalysts. On top of that, the CPI‐SA shows much higher threshold energy (E_0_) (11 217.1 eV) as compared to Ir‐foil (11 215.8 eV) in the first derivative of the XANES spectrum (Figure 2b), suggesting the depletion of Ir‐d band.^[^ ^21^ ^]^ Such a depletion in the d‐band along with the high surface oxygen chemisorption (corresponding to the high H_A_) are typical features of single atoms, indicating Ir is present in the form of single atoms in CPI‐SA.^[^ ^29^ ^]^ Figure 2c demonstrates the Fourier‐transformed extended X‐ray absorption fine structure (FT‐EXAFS) spectra of the CPI catalysts and the reference samples at Ir L_3_‐edge, while the corresponding quantitative structure parameters are summarized in **Table** **1** and the overlay of fitting curves and experimental spectra are compared in Figure S4 (Supporting Information). Accordingly, the Ir‐foil shows a prominent peak C at 2.58 Å ascribed to the Ir─Ir scattering path. The absence of Ir─Ir scattering and the coordination number (CN) for the Ir─Ir bond pair (CN_Ir‐Ir_ = 0) in the CPI‐SA spectrum (Table 1) strongly confirms the formation of Ir single atoms in CPI‐SA. Meanwhile, consistent with the high H_A_ in the XANES spectra, the presence of CN for the Ir‐O bond pair (CN_Ir‐Ir_ = 2.91) confirms the severe oxidation of Ir single atoms. Besides, the existence of the Ir─Ir bond pair (CN_Ir‐Ir_ = 2.55) confirms the formation of sub‐nano metallic Ir clusters in CPI‐NC and is consistent with former STEM findings. For CPI‐NP (see the blue line), radial peaks across 1.21–23.5 Å are attributed to Ir‐O (CN_Ir‐O_  =  2.23), Ir─Pd (CN_Ir‐Pd_  =  2.41) and Ir─Ir (CN_Ir‐Ir_  =  2.56) bond pairs. Herein, it's worth noticing that the CPI‐NP shows nearly similar CN for the Ir─Ir bond pair as that of CPI‐NC and seems controversial, however, can be attributed to the significant extant of Ir─Pd heteroatomic intermixing (CN_Ir‐Pd_  =  2.41). Given that the wavelet transform (WT) patterns can discriminate the backscattering atoms by k‐space resolution along with radial distance resolution and therefore WT analysis of FT‐EXAFS spectra at Ir L_3_ edge is performed to further confirm the atomic Ir dispersion.^[^ ^30^ ^]^ As shown in Figure 2d, the absence of intensity maxima at 2.6 Å (corresponds to the Ir─Ir bond pair in Ir foil) again confirms the formation of single Ir‐atoms in CPI‐SA. Meanwhile, the CPI‐SA shows an intensity maxima at 1.6 Å (corresponds to the Ir‐O bond pair in Ir‐CNT)^[^ ^29^ ^]^ suggesting the severe oxidation of Ir‐ single atoms. Consistent with FT‐EXAFS results, the CPI‐NC and CPI‐NP catalysts exhibit the intensity maxima for both Ir‐O and Ir─Ir bond pairs, where the intense maxima in the WT pattern of CPI‐NP can be attributed to additional contribution from Ir─Pd scattering path and in good agreement with the FT‐EXAFS results.

**Table 1:** Quantitative results of X‐ray absorption spectroscopy model analysis of CPI catalysts and control samples at Ir L_3_‐edge, Pd K‐edge, and Co K‐edge.

Sample	Ir L_3_‐edge			Pd K‐edge			Co K‐edge		
	bond pair	CN	χ (%)	bond pair	CN	χ (%)	bond pair	CN	χ (%)
CPI‐SA	Ir─Ir	0	N/A	Pd─Pd	6.18	84.7	Co─Co	2.85	N/A
	Ir─O	2.91		Pd─Ir	1.12	15.3	Co─O	3.61	
	Ir─Pd	0		Pd─Co	0	0	Co─O^2^	0	
	CN_total_	2.91		CN_total_	7.3		CN_total_	6.46	
CPI‐NC	Ir─Ir	2.55		Pd─Pd	6.07	80.9	Co─Co	1.66	
	Ir─O	3.43		Pd─Ir	1.43	19.1	Co─O	3.12	
	Ir─Pd	0		Pd─Co	0	0	Co─O^2^	0	
	CN_total_	5.98		CN_total_	7.5		CN_total_	4.78	
CPI‐NP	Ir─Ir	2.56		Pd─Pd	6.02	65.4	Co─Co	2.07	
	Ir─O	2.23		Pd─Ir	3.19	34.6	Co─O	3.12	
	Ir─Pd	2.41		Pd─Co	0	0	Co─O^2^	0	
	CN_total_	7.2		CN_total_	9.21		CN_total_	5.17	
Co@Pd	N/A			Pd─Pd	6.81	100	Co─Co	2.88	
				Pd─Ir	0	00	Co─O	3.42	
				Pd─Co	0	0	Co─O^2^	0	
				CN_total_	6.81		CN_total_	6.3	
Ir‐CNT	Ir─Ir	3.94		N/A					
	Ir─O	0							
	Ir─Pd	0							
	CN_total_	3.94							
Pd‐CNT	N/A			Pd─Pd	7.71	100	N/A		
				Pd─Ir	0	0			
				Pd─Co	0	0			
				CN_total_	7.71				
Co‐CNT	N/A						Co─Co	0.91	N/A
							Co─O	1.64	
							Co─O^2^	1.41	
							CN_total_	3.96	

Figure 2e compares the XANES spectra of the CPI catalysts and the reference samples (Pd‐CNT and Co@Pd) at the Pd K‐edge, where peaks M and N correspond to the oxidized and metallic states of Pd, respectively.^[^ ^31^ ^]^ Notably, the intense peak N along with a similar position of inflection point (I) and the peaks (Y and Y*) in the first deviation curve (Figure 2f) confirms the metallic characteristics of Pd in CPI catalysts and reference samples. The FT‐EXAFS spectra of the CPI catalysts and the reference samples are compared in Figure 2g, while the corresponding structural parameters are summarized in Table 1 and the overlay fitting curves with the experimental spectra are provided in Figure S5 (Supporting Information). Accordingly, the absence of Co intermixing (χ(Co)) confirms the formation of Pd NPs over the Co‐oxide surface. Notably, as compared to Pd‐CNT, the Co@Pd exhibits a suppressed radial peak, which refers to the reduced CN for the Pd─Pd bond pair (CN_Pd‐Pd_ = 6.81) and confirms the presence of local surface defects on Pd crystal in the Co@Pd NC. For CPI catalysts, the Ir intermixing in Pd crystal (χ(Ir)) is increased from 15.3 to 34.6% with the Ir content rising from 1.0 to 7.0 wt.% and can be consistently confirmed by the progressively suppressing intensity of maxima at 2.51 Å (corresponding to the Pd─Pd bond pair) in the WT patterns (Figure 2h). Furthermore, compared to that of Co@Pd, the increased total coordination number (CN_total_) with the decreased diffraction peak intensity in the XRD pattern (Figure S2, Supporting Information) indicates the improved local structure ordering around Pd atoms with the suppressed crystallinity in the CPI‐SA and CPI‐NC catalysts. These scenarios seem controversial with each other, however, can be rationalized by the decoration of Ir atoms in the defect sites and their subsequent oxidation on the Co@Pd NC surface. Compared to the structure of CPI‐NC, a substantially increased CN_total_ by 1.71 (from 7.5 to 9.21) and χ(Ir) by 15.5% (from 19.1 to 34.6%) respectively depict the improved local structure ordering around Pd atoms and the formation of IrPd alloy in CPI‐NP catalyst.

The local structure evolutions of the Co‐crystal in CPI catalysts and reference samples were unveiled by XAS analysis at Co‐K‐edge. Figure 2i shows the normalized Co K‐edge XANES spectra of CPI catalysts compared with Co@Pd and Co‐CNT. In a Co K‐edge spectrum, the pre‐edge (peak R) and the white line intensities (H_G_), respectively, refer to the local symmetry around Co atoms and the extent of unoccupied states in the 4p orbitals; whereas the position of the *I* (peaks Z* and Z’ in Figure 2j) corresponds to the oxidation state of Co‐atoms.^[^ ^31^ ^]^ As shown in the inset (green region) of Figure 2i, compared to that of Co‐CNT, the substantially suppressed pre‐edge intensity and enhanced H_A_ are indications for the local distortion around the Co‐atoms and the charge relocation from the Co 4p orbital to neighboring atoms in Co@Pd and CPI catalysts, respectively. An even closer inspection of pre‐edge and white line intensities reveals that CPI‐SA exhibits the lowest pre‐edge and highest white line intensities, resembling the highest amount of charge transfer from Co to neighboring atoms. Moreover, quantitative local atomic structure analysis around the Co‐sites was further elucidated by model analysis of FT‐EXAFS spectra (the overlay fitting curves and experimental spectra are provided in Figure S6, Supporting Information). As shown in Figure 2k, the presence of radial peaks across 1.9 to 2.1 Å reveals the metallic characteristics with a CN of 0.91 for the Co‐Co bond pair in Co‐CNT (Table 1), while the broad radial peaks centered at 1.6 and ≈2.2 Å resemble the formation of an amorphous surface Co oxide. The two components are locally disordered due to that their coordination numbers are substantially smaller than that of the standard crystal model (CN_Co‐O_ = 6, CN_Co‐Co_* = 6, CN_Co‐O2_ = 6). In Co@Pd and CPI catalysts, the radial peaks J (at ≈2.060 Å) and K (3.129 Å) are contributions from Co─O and Co─Co bond pairs, respectively, in the Co_3_O_4_ oxides. More specifically, the CN_Co‐O_ is 3.42 and the CN_Co‐Co_ is 2.88 in Co@Pd. As similarly depicted in the Pd K‐edge results, compared to the standard crystal structure, the small CN for the two bond pairs suggests the formation of short‐range ordered Co oxides. In the meantime, a sharp pre‐edge hump indicates that the Co sites are retained in tetrahedral symmetry. As compared to the EXAFS profiles of Co@Pd, the offset (to the left) accompanied by a certain extent of broadening in radial peaks depicts the relocation of Co atoms in the CPI‐SA catalyst. Such a restructure could be attributed to the galvanic replacement of Co atoms by interacting with Ir^3+^ ions and redistribution of Co^2+R^ (residual Co^2+^ in solution) by the interaction with a reducing agent (NaBH_4_). Compared to the local structure of CPI‐SA, a dramatic decrease of CN in Co─O and Co‐Co respectively by 0.49 and 1.19 indicates the local disordering by the severe extent of the aforementioned two relocation pathways around Co atoms in CPI‐NC. That structural information reveals the direct contact of Co oxide to the reaction system thus rationalizing the formation of the un‐conformal Pd layer on the Co oxide surface. Increasing local ordering around Co atoms reveals the suppression of galvanic replacement by Ir ions. Such a scenario can be rationalized by the heterogeneous nucleation and crystal growth of IrPd alloy and surface oxidation by further increasing Ir contents to 7.0 wt.%. These observations are complementarily confirmed by the WT analysis in Figure 2l.

### 2.2 Electrochemical characterizations

**Figure 3:**
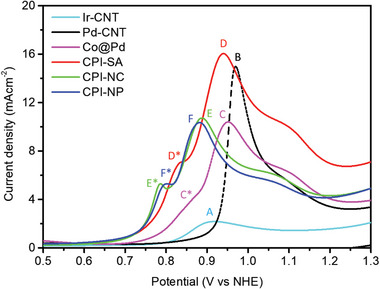
CO stripping voltammograms of Ir‐CNT, Pd‐CNT, Co@Pd, and CPI catalysts.

CO‐stripping analysis (**Figure** **3**) has been employed to elucidate the surface chemical identities of CPI catalysts and reference samples. Typically, the positions of adsorbed CO (i.e., CO^ads^) oxidation peaks within a CO‐stripping curve provide insights into the necessary potential for CO oxidation.^[^ ^32^ ^]^ Meanwhile, the area beneath the CO oxidation peak is indicative of the density of surface‐active sites that have undergone CO chemisorption.^[^ ^33^ ^]^ It is evident from Figure 3 that Ir‐CNT shows a broad and suppressed CO‐oxidation peak A across ≈ 0.75 to 1.1 V versus NHE, indicating the relatively poor selectivity of CO^ads^ oxidation due to the insignificant binding energy differences between sorption sites at open and compact sites of Ir‐CNT. Compared to Ir‐CNT, Pd‐CNT exhibits a sharp peak B at ≈ 0.97 V (volt vs NHE) with a positive offset of 55 mV, suggesting the stronger selectivity between opened and compact facets and the higher energy barrier for CO^ads^ oxidation. For the Co@Pd NC, an offset of main CO oxidation peak C (≈0.950 V vs NHE) by 20 mV suggests a reduced energy barrier for CO^ads^ oxidation as compared to Pd‐CNT. Such a significant decrease in the energy barrier can be attributed to the severe electron relocation from Co‐to‐Pd (consistent with former XAS findings) due to strong electronegativity difference and lattice strain. Moreover, the presence of a shoulder peak C* (≈ 0.865 V vs NHE) refers to the current contribution from the CO^ads^ oxidation at the high density of low energy barrier sites and higher‐order open facets of the Pd‐crystals.^[^ ^31^ ^]^ Meanwhile, CPI‐SA manifests a significantly enhanced peak D with the largest peak area and an offset of10 mV (compared to the position of peak B in Co@Pd NC), respectively, revealing the highest density of reaction sites and lower energy barrier for CO^ads^ oxidation on Co@Pd NC after the decoration of Ir single atoms. Besides, a pronounced peak D* with an offset of −0.03 V (compared to the peak C* of Co@Pd) corresponds to the current contribution from the CO^ads^ oxidation at the high density of low energy barrier open (i.e., (200) and (220)) facets. Unsurprisingly, when Ir‐content is raised to 3 wt.% (i.e., CPI‐NC) and 7 wt.% (i.e., CPI‐NP), the positions of the main CO^ads^ oxidation peaks E and F are shifted to lower potentials and achieved nearly similar values as that of Ir‐CNT (peak A), which is attributed to the higher extent of Ir characteristic and reduced binding energy for CO^ads^ oxidation on the compact facet. Meanwhile, the dramatically suppressed intensities (by 45% compared to Peak D in CPI‐SA) of the main CO^ads^ oxidation peaks (i.e., peaks E and F) indicate the reduced density of reaction sites on CPI‐NC and CPI‐NP surface relative to CPI‐SA. Moreover, the intense peaks E* and F*, respectively, for CPI‐NC and CPI‐NPs indicate the relatively high sensitivity toward CO^ads^ oxidation at open facets and Ir─Pd sites (i.e., local alloy of Ir─Pd).

**Figure 4:**
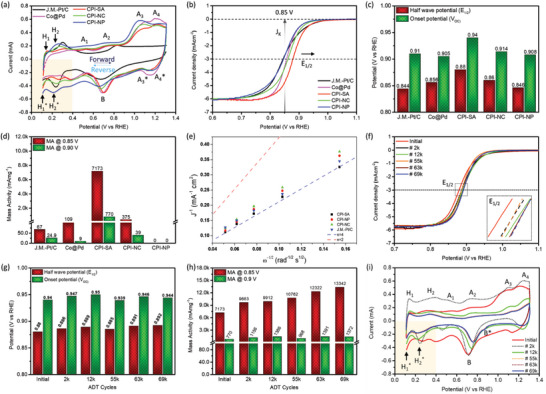
Electrochemical results of CPI catalysts. a) CV, b) LSV curves, c) corresponding onset potential (*V*_OC_) and half‐wave potential (*E*_1/2_), d) ORR mass activity at 0.85 (vs RHE) and 0.90 V (vs RHE), and e) Koutecky–Levich (K.L.) plot of CPI catalysts compared with Co@Pd and commercial J.M.‐Pt/C catalysts. f) LSV and g) corresponding onset potential (*V*_OC_) and half‐wave potential (*E*_1/2_), h) mass activities at 0.85 and 0.9 V versus RHE, and i) CV curves of CPI‐SA at pristine conditions and selected ADT cycles.

The ORR performances of CPI catalysts are assessed by cyclic voltammetry (CV) and linear sweep voltammetry (LSV) analysis. The commercial J.M.‐Pt/C and Co@Pd were also tested under the same conditions and compared for reference. As shown in **Figure** **4**a, the Co@Pd exhibits a smeared peak profile (forward sweep) in the underpotential deposition (H_UPD_) region (similar to that of Pd‐CNT (Figure S7a, Supporting Information)) with the pair of peaks (A_3_/A_4_ and A_3_*/A_4_*; similar to Co‐CNT) in the potential range of 0.9 to 1.3 V versus RHE, respectively, indicating the formation of Pd NPs over Co‐oxide support. Besides, compared to Co@Pd, it is interesting to observe that the CPI‐SA exhibits a pair of significantly sharp and narrow peaks (H_1_/H_1_*) at ≈ 0.1 V versus RHE in the H_UPD_ region, which is attributed to the strong H_2_ evolution activity.^[^ ^34^ ^]^ As widely discussed in the literature, such kind of strong H_2_ evolution activity is a typical trademark of the atomic species,^[^ ^35^, ^36^ ^]^ consistently confirming the presence of Ir single atoms on the Co@Pd surface. Moreover, the current responses (A_1_–A_4_) in the forward sweep are due to the formation of oxide species on the surface of different combinations of Co, Pd, and Ir, while the obvious peak B in the backward sweep is corresponding to the reduction of oxide species from Pd surface.^[^ ^37^ ^]^ The corresponding potential of the oxide reduction peak is strongly associated with the binding energy of oxygen species.^[^ ^16^ ^]^ Remarkably, the CPI‐SA showed the highest positive ORR onset potential (≈0.910 V vs RHE), suggesting the lowest energy barrier. Besides, the oxide reduction peaks for CPI‐NC and CPI‐NP are dramatically downshifted in terms of intensity and potential, respectively, indicating the reduced density of reaction sites and increased energy barrier for oxide reduction. These observations are in good agreement with CO‐stripping results. Moreover, the oxide reduction and H_UPD_ region peak profiles of CPI‐NP are nearly similar to that of Ir‐CNT (Figure S7a, Supporting Information), advocating that decorated Ir‐species are present in the form of nanoparticles in CPI‐NP, which is consistent with former STEM findings.

Figure 4b compares the ORR polarization curves of CPI catalysts with commercial J.M.‐Pt/C and Co@Pd, where the current densities were normalized by the area of the carbon electrode. Notably, the CPI‐SA exhibits the highest half‐wave potential (*E*_1/2_) and onset potential (*E*_OC_), suggesting the lowest energy barrier and high reaction kinetics for ORR. Besides, *E_1/2_* and *E*_OC_ values are progressively decreased when Ir‐loading goes up (Figure 4c). Such a reverse trend in *E_1/2_* and *E*_OC_ is consistent with the position of the oxide reduction peak in CV curves (Figure 4a). To further assess the ORR activity, mass activities (MA) of CPI catalysts were obtained via normalizing the kinetic current densities (denoted as *J*_k_) at 0.85 and 0.90 V versus RHE with respect to the Ir‐loading (detailed procedure for mass activity calculation is given in Note S12, Supporting Information).^[^ ^16^ ^]^ Unsurprisingly, the CPI‐SA demonstrates an outstanding MA of 7173 and 770 mAmg_Ir_^−1^ at 0.85 and 0.90 V versus RHE, respectively (Figure 4d). Compared to the commercial J.M.‐Pt/C catalyst (67 and 24.9 mAmg_Pt_^−1^ at 0.85 and 0.90 V versus RHE), these MA values are improved by ≈107 and ≈31‐folds at 0.85 and 0.90 V versus RHE, respectively. Besides, a sharp downfall in MA of CPI‐NC and CPI‐NP can be attributed to the increasing dimension of the Ir species. For clarification, the results of reference samples (Co‐CNT, Pd‐CNT, Ir‐CNT, Co@Pd, Co@Ir, and Pd@Ir) are compared in the Figure S7 and Table S4 (Supporting Information). Accordingly, the J_k_, *E_1/2_*, and *E*_OC_ of the reference samples are far below as compared to that of CPI‐SA, consequently proving the reaction kinetics of the CPI‐SA is dominated by the Ir single atoms. Finally, the ORR performance of CPI‐SA is compared with the literature (Table S5, Supporting Information), where the CPI‐SA is superior to other catalysts. Moreover, the nearly parallel Koutecky–Levich (K.L.) plots suggest a 4‐electron transfer pathway and first‐order reaction kinetics toward the concentration of dissolved oxygen (O_2_) at different potentials (Figure 4e).

The CPI‐SA not only demonstrated an outstanding performance but robust long‐term durability as well in ORR. According to the representative ORR polarization curves (Figure 4f), the CPI‐SA shows a positive shift (+12 mV) in the *E_1/2_* (Figure 4g) accompanied by an exceptional MA of 13 342 and 1372 mAmg_Ir_^−1^ respectively at 0.85 and 0.90 V versus RHE (Figure 4h), elucidating that the durability of the CPI‐SA in ADT is not limited up to 69k cycle. This phenomenon can be attributed to the surface restructure (redistribution of Ir single atoms and removal of surface oxide from the Pd surface) and is consistently proved by the CV analysis at the selected ADT cycles (Figure 4i). Accordingly, the position of the oxide reduction peak (B) in the backward sweep progressively shifted to the higher potentials with increasing ADT cycles, reflecting the lower energy barrier for ORR. Herein it's worth noticing that the position and intensity of the oxide reduction peak “B” are stable after 55k ADT cycles, while peaks H_1_/H_1_* are still survived. More interestingly, an additional peak B* (≈ 0.870 V vs RHE) is evolved, corresponding to the oxide reduction from single Ir atoms with lower energy barriers compared to that of neighboring Pd‐sites. These observations concluded that the oxygen reduction activity is mainly dominated by Ir single atoms at the catalyst surface at that time, instead of the initial Pd phase.

### 2.3 In situ Partial Fluorescence Yield (PFY) Mode XAS Inspections

**Figure 5:**
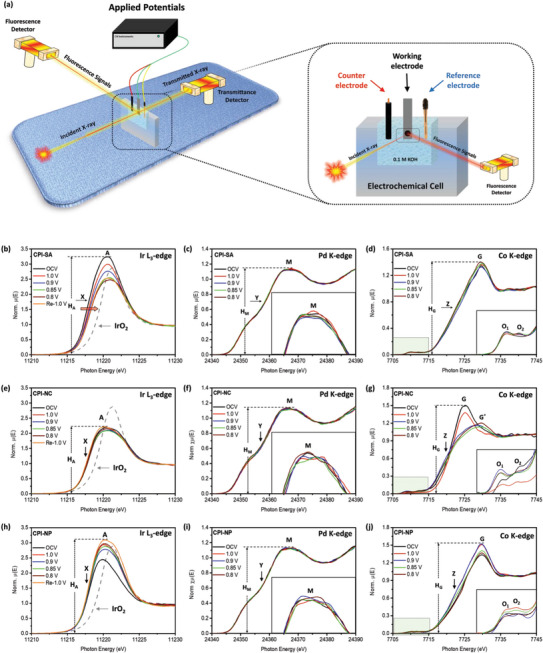
a) The schematic representation of the in situ PFY mode XAS experiment setup and the in situ electrochemical cell. In situ PFY‐XANES spectra of CPI‐SA at b) Ir L_3_‐edge, c) Pd K‐edge, and d) Co K‐edge. In situ PFY‐XANES spectra of CPI‐NC at e) Ir L_3_‐edge, f) Pd K‐edge, and g) Co K‐edge. In situ PFY‐XANES spectra of CPI‐NP at h) Ir L_3_‐edge, i) Pd K‐edge, and j) Co K‐edge. The in situ XAS data were collected in the O_2_ saturated alkaline electrolyte solution of 0.1 m KOH (pH = 13) at the OCV condition and applied potentials of 1.0 to 0.8 V versus RHE.

The performance descriptors and corresponding ORR pathways on the surface of CPI‐SA have been assessed via in situ PFY mode XAS inspections at Ir L_3_‐edge, Pd K‐edge, and Co K‐edge. The in situ PFY mode XAS analysis was carried out in fluorescence mode by using a customized electrochemical cell integrated with a standard three‐electrode electrochemical workstation (**Figure** **5**a).^[^ ^31^, ^38^ ^]^ The in situ Ir L_3_‐edge PFY‐XANES spectra of CPI‐SA under potential‐driven conditions (the applied potentials were chosen as per the ORR polarization curves in Figure 4b) are shown in Figure 5b. Accordingly, the progressively suppressed white line intensity (H_A_) to the maximum indicates the increasing occupied density of the Ir‐5d orbital and can be attributed to the electron localization from neighboring atoms to the Ir‐atoms with increasing potentials from 1.0 to 0.8 V versus RHE.^[^ ^38^ ^]^ With the unchanged profile (inflection point Y) of in situ PFY‐XANES spectra at the Pd K‐edge (Figure 5c), the electron relocation from Co atoms is confirmed and is consistently proved by the results of the in situ PFY‐XANES at the Co K‐edge (Figure 5d). On top of that, considering the progressively suppressing H_A_ with increasing applied potentials (i.e., the higher extent of electron relocation on Ir atoms), the positive shift of inflection point X and the first derivative peak in Figure S8a (Supporting Information) (i.e., the higher oxidation (valence) state of Ir‐atoms) is controversial. However, such a scenario can be attributed to further electron transfer from Ir‐atoms (localized electrons from neighboring sites) to the adsorbed oxygen species (O^ads^) during O_2_ splitting (i.e., O_2_ →2 O^ads^).^[^ ^28^ ^]^ These scenarios unambiguously elucidate that the Ir single atoms are the active center of O_2_ splitting under potential‐driven conditions. More importantly, taking into account that O_2_ splitting is taking place on Ir‐sites, the lower extent of O^ads^ (suppressed H_A_ under potential‐driven conditions) on the Ir atoms can be attributed to the faster reaction kinetics of O_2_ splitting (i.e., O_2_ → 2O^ads^) and subsequent relocation of O^ads^ to neighboring sites for hydration step (O^ads^ + H_2_O + 4e^−^ → 4OH^−^). Such a scenario is confirmed by reversing the applied voltage. Compared to 0.8 V, the increased H_A_ at Re‐1.0 V condition (i.e., the applied potential was reversed at 1.0 V vs RHE) indicates the presence of O^ads^ on the Ir‐atoms, suggesting that O^ads^ relocation kinetics is slow at lower applied potentials as compared to higher potentials. Furthermore, the in situ PFY‐XANES spectra of CPI‐SA at Pd K‐edge (Figure 5c) show the unchanged inflection point Y (corresponding to the peaks Y and Y* in the first derivative curve (Figure S8b, Supporting Information)), suggesting the stable valence state (absence of electron relocation) of Pd atoms under potential‐driven conditions. However, complimentary with the broadened current response (compared to Co@Pd) in the double layer region of CV curves (Figure 4a) and the strong electron relocation around Ir‐single atoms on Pd NPs, the pronounced peaks M (inset of Figure 5c) at the applied potential of 1.0 V versus RHE can be attributed to the presence of OH^−^ ions (O^ads^ + H_2_O + 4e^−^ → 4OH^−^) on the Pd atoms, indicating that the Pd sites favor the hydration step, where relocated O^ads^ atoms reduce in OH^−^ ion. Moreover, consistent with H_A_ in the Ir L_3_‐edge spectra, the reduced H_M_ in the Pd K‐edge spectra at higher potentials confirms the faster reaction kinetics for the desorption of OH^−^ ions from the Pd surface. Finally, compared to the OCV condition, the increased H_G_ in the PFY‐XANES spectra of the Co K‐edge (Figure 5d) confirms the substantial charge relocation from Co atoms under potential‐driven conditions. Figure 5e shows the in situ PFY‐XANES spectra of CPI‐NC at Ir L_3_‐edge, where the Ir‐atoms show an unchanged inflection point (corresponding to the peak X in the first derivative curve (Figure S8d, Supporting Information)), suggesting the stable chemical state of Ir in CPI‐NC under potential‐driven conditions. Moreover, compared to the Ir L_3_‐edge spectra of CPI‐SA, the CPI‐NC exhibits the suppressed H_A_ (high density of occupied state in the 5d orbital) and insignificant variation with increasing potentials (Figure 5e), which can be attributed to the sluggish reaction kinetics of O_2_ splitting due to the increased dimension of Ir‐species and consistent with the ORR performance of CPI‐NC. Meanwhile, the small variation in the peak profiles of Pd K‐edge PFY‐XANES spectra (Figure 5f) can be attributed to the accumulation of OH^−^ ions on the Pd surface due to suppressed O_2_ splitting on Ir‐domains. More interestingly, a significant variation in the Co K‐edge PFY‐XANES spectra of CPI‐NC is observed under potential‐driven conditions. As shown in Figure 5g, the suppression of H_G_ to the maximum extent along with the peak shift to higher energy values (peak G*) with the applied potential from 1.0 to 0.85 V versus RHE, suggesting the removal of surface chemisorbed oxygen (O^ads^) and the reduction of Co‐oxide, respectively. Considering the absence of electron relocation (i.e., insignificant variations in the in situ PFY‐XANES spectra of CPI‐NC at Ir‐L_3_ edge and Pd K‐edge) from Co‐atoms, such a scenario is obvious under potential‐driven conditions. An even closer inspection of Co K‐edge PFY‐XANES spectra reveals the increased H_G_ after further increasing the applied potential from 0.85 to 0.8 V versus RHE and can be attributed to the presence of abundant O^ads^ (due to sluggish O_2_ kinetics at Ir domains) on Co‐atoms. For CPI‐NP, the abruptly increased H_A_ (Figure 5h) and H_G_ (Figure 5j) under potential‐driven conditions, respectively, in the Ir L_3_‐edge and Co K‐edge PFY‐XANES spectra suggest the presence of a high extent of O^ads^ on the active sites. Complimentary with the unchanged valence state of Pd‐atoms (Figure 5i), these scenarios confirm the chemically inert nature of CPI‐NP for ORR and are in good agreement with its ORR performance (Figure 4d). For further clarification, results of the corresponding PFY‐XANES analysis at Pd K‐edge (Figure S9a, Supporting Information) and Co K‐edge (Figure S9b, Supporting Information) of Co@Pd are given, while the in situ PFY‐XANES spectra of Ir‐CNT (Figure S10a, Supporting Information) and IrO_2_ (Figure S10b, Supporting Information) are demonstrated for reference.

**Figure 6:**
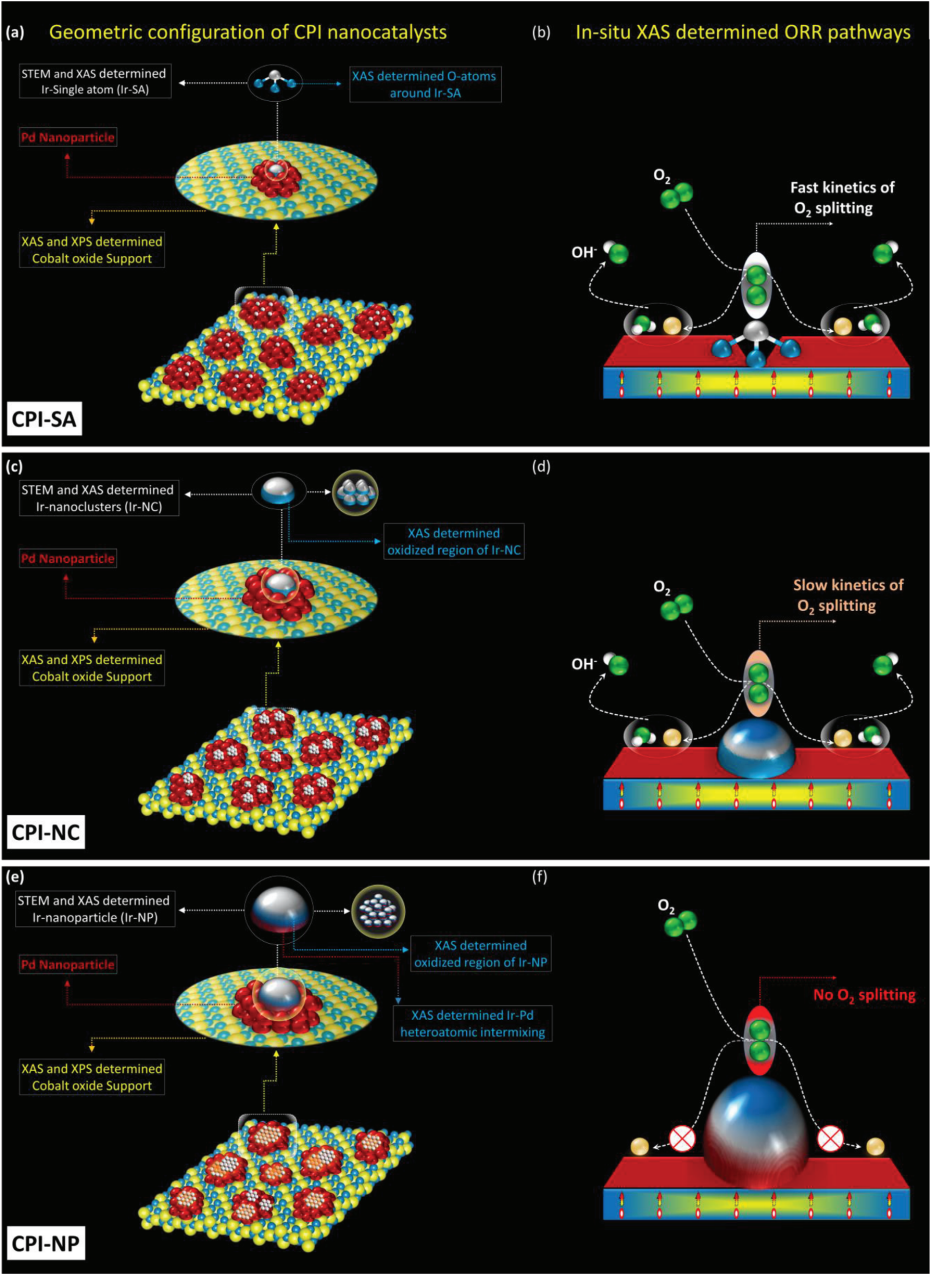
The schematic geometric configuration of a) CPI‐SA, c) CPI‐NC, e) CPI‐NP and corresponding ORR pathways of b) CPI‐SA, d) CPI‐NC, f) CPI‐NP.

Integrating the findings from both physical and electrochemical characterizations along with in situ PFY‐XANES analysis allows for a detailed understanding of the catalyst's structure and sheds light on the oxygen reduction reaction (ORR) pathways. The resulting insights into the catalyst's atomic arrangement and the corresponding ORR pathways are visually represented in **Figure** **6**. As shown in Figure 6a, the CPI‐SA consisting of the Ir‐SAs on the cobalt‐oxide‐supported Pd NPs, where the Ir‐SAs promote the O_2_ splitting (O_2_ → 2O^ads^), while Pd NPs favor the subsequent hydration step (O^ads^ + H_2_O + 4e^−^ → 4OH^−^) in ORR (Figure 6b). Figure 6c,d, respectively, demonstrate the geometric configuration of CPI‐NC and corresponding ORR pathways. Accordingly, Ir‐NCs are partially oxidized and therefore the O_2_ splitting reaction kinetics is relatively suppressed. For CPI‐NP (Figure 6e), the Ir‐species are grown in the form of NPs on the surface of Pd NPs. In this case, due to increased Ir metal content, the severe galvanic replacement reaction is expected between Ir and Pd atoms (Ir^3+^ + Pd^0^ → Ir^0^ + Pd^2+^) and therefore some extent of heteroatomic intermixing is observed in CPI‐NP catalyst. Unsurprisingly, consistently confirmed by the in situ PFY‐XANES, due to the severe surface oxidation the Ir‐NPs are inert for O_2_ splitting (Figure 6f).

## 3 Conclusions

Besides significant strides in the development of heterogeneous single‐atom catalysts (SAC)s, their activity and stability are severely limited by the lack of ensemble sites and susceptibility to aggregation, oxidation, and other structural changes, respectively. This study addresses the aforementioned challenges associated with single‐atom catalysts. In this context, a novel heterogeneous catalyst comprising Ir‐single atoms decorated Pd nanoparticle on the cobalt‐oxide support (denoted as CPI‐SA) is demonstrated. As‐prepared CPI‐SA catalyst exhibits remarkably high‐mass activity of 7173 and 770 mAmg_Ir_^−1^, respectively, at 0.85 and 0.90 V versus RHE toward ORR in the alkaline medium, which not only outperforms the previously reported Ir‐based catalysts but also surpass the commercial J.M.‐Pt/C (20 wt.% Pt) catalyst by more than ≈107‐fold (at 0.85 V vs RHE). Of special relevance, this catalyst exhibits exceptional stability and maintains its 186% MA (13342 mAmg_Ir_^−1^ @ 0.85 V vs RHE.) as that of the initial condition when operated in the accelerated degradation test (ADT) for 69 000 cycles (3 months). We believe that obtained insights can serve as a valuable guide for the design of advanced catalysts, promoting the deliberate combination and synergy of multiple active species to further advance the field of catalysis.

## 4 Experimental Section

### Preparation of Ternary Catalysts

The synthesis of Co─Pd─Ir (CPI) ternary catalysts was accomplished through a meticulously controlled process involving ion chemisorption, wet chemical reduction method, and ambient annealing.^[^ ^16^ ^]^ Specifically, the size and distribution of Ir‐species were carefully controlled by regulating the molar ratios of Ir/Pd and the reaction time of the Ir decoration. Henceforth, the CPI catalysts with the surface decoration of Ir‐SAs, Ir‐NCs, and Ir‐NPs are denoted as CPI‐SA, CPI‐NC, and CPI‐NP, respectively. The detailed synthesis procedure is described in Note S1 (Supporting Information). The cobalt‐oxide‐supported Pd NPs (denoted as Co@Pd) without surface decoration of Ir‐species were also prepared for fair comparison.

## Conflict of Interest

The authors declare no conflict of interest.

## Supporting information

Supporting Information

## Data Availability

The data that support the findings of this study are available from the corresponding author upon reasonable request.
