# Supplementary material for: Iridium Single Atoms to Nanoparticles: Nurturing the Local Synergy with Cobalt‐Oxide Supported Palladium Nanoparticles for Oxygen Reduction Reaction
Source: Adv Sci (Weinh). 2024 Jun 27;11(33):2404076. doi: 10.1002/advs.202404076 (PMC11434211; doi:10.1002/advs.202404076)
Supplement: Supplementary file 1 — Supporting Information [file ADVS-11-2404076-s001.docx]

Supporting Information for

**Iridium Single Atoms to Nanoparticles: Nurturing the Local Synergy with Cobalt-Oxide Supported Palladium Nanoparticles for Oxygen Reduction Reaction**

Dinesh Bhalothia,^a^ Che Yan,^a^ Nozomu Hiraoka,^b^ Hirofumi Ishii,^b^ Yen‑Fa Liao,^b^ Sheng Dai,^c^ Po-Chun Chen*^d^ and Tsan-Yao Chen*^a^

Affiliations:

^a.^ Department of Engineering and System Science, National Tsing Hua University, Hsinchu 30013, Taiwan.

^b.^ National Synchrotron Radiation Research Center, Hsinchu 30076, Taiwan

^c.^ School of Chemistry & Molecular Engineering, East China University of Science and Technology, Shanghai 200237, P.R. China

^d.^ Department of Materials and Mineral Resources Engineering, National Taipei University of Technology, Taipei 10608, Taiwan

*Corresponding Author:

Po-Chun Chen

Department of Materials and Mineral Resources Engineering,

National Taipei University of Technology,

Taipei 10608, Taiwan

Email: cpc@mail.ntut.edu.tw

FAX: +886-3-27317185

Prof. Tsan-Yao Chen

Department of Engineering and System Science,

National Tsing-Hua University,

Hsinchu 300, Taiwan

Email: chencaeser@gmail.com;

FAX: +885-3-5720724

1. **Sample preparation**

The Co-Pd-Ir (CPI) ternary catalysts were synthesized via a three-step self-aligned wet chemical reduction method with proper sequence and reaction time controls in heterogeneous nucleation and crystal growth processes of the three elements (**Scheme S1**). Prior to synthesis, the surface functionalization of catalyst support (i.e. multi-walled carbon nanotube (MWCNT, Cnano Technology Ltd.)) has been done via acid treatment for strengthening the attachment of metallic crystals on their surface. The acid treatment was processed in 4.0 M H_2_SO_4_ at 80℃ for 6 hours followed by washing in deionized (D.I.) water until the pH value of rinsing water is 6.0. For preparing CNT supported Co oxide, 1.2 g of 5.0 wt% CNT solution in D.I. water (i.e., containing 60 mg of CNT powder) is dispersed in 3.06 g of an aqueous solution containing 0.1 M cobalt (II) chloride (CoCl_2_, 99%, Sigma-Aldrich Co.) and stirred at 200 rpm for 4 h (**step-1**). As-prepared mixture (Co^2+^ adsorbed on CNT surface; Co^2+_ads^-CNT) contains 0.306 mmoles (18 mg) of Co^2+^ ions with a weight ratio of 30 wt.% for Co/CNT. In **step-2**, a 5.0 ml of D.I. water solution consisting of 0.11 g of sodium borohydride (NaBH_4_; 99%, Sigma-Aldrich Co.) was instantly dropped into the solution prepared in the 1^st^ step (i.e. Co^2+_ads^-CNT) and stirred at 200 rpm for 10 seconds. In this step, metastable Co metal nanoparticles were formed (i.e. Co-CNT), which later turned into Co-oxide (i.e. CoO_x_). Subsequently, 3.06 g of Pd precursor solution containing 0.306 mmoles of Pd metal ions (i.e. 0.1 M) was added into Co/CoO_x_-CNT solution to grow the Pd-NPs on Co/CoO_x_ support (namely Co@Pd). In this step, Pd^2+^ ions are reduced by the excessive amount of NaBH_4_ added in **step-2** and deposited on Co/CoO_x_ surface. For preparing the Pd precursor solution, palladium chloride (PdCl_2_, 99%, Sigma-Aldrich Co.) was dissolved in 1.0 M of HCl_(aq)_. The molar ratio of Pd/Co is 1.0 in Co@Pd NPs. Following the synthesis of Co@Pd NPs, an appropriate amount of Ir-precursor solution was added into the solution containing Co@Pd NPs (**step 4**). Prior to the surface decoration of Ir, the as-prepared Co@Pd NC were subjected to ultrasonication treatment for creating sub-nano defects for anchoring the Ir-species in different dimensions. The Ir-precursor solution was prepared by dissolving 1.0 g of Iridium (III) chloride (IrCl_3_; 99%, Sigma-Aldrich Co.) in 18.36 of D.I. water (i.e. 0.1 M solution). For controlling the size and distribution of Ir-decoration, different molar ratios of Ir/Pd is set to prepare CPI catalysts with different Ir-content (i.e. 1.0, 3.0 and 7.0 wt.%). In the following of this article, the Ir clusters decorated Co@Pd NPs are denoted as CPI-SA, CPI-NC and CPI-NP for samples with 1.0, 3.0 and 7.0 wt.% Ir-loading, respectively.

**
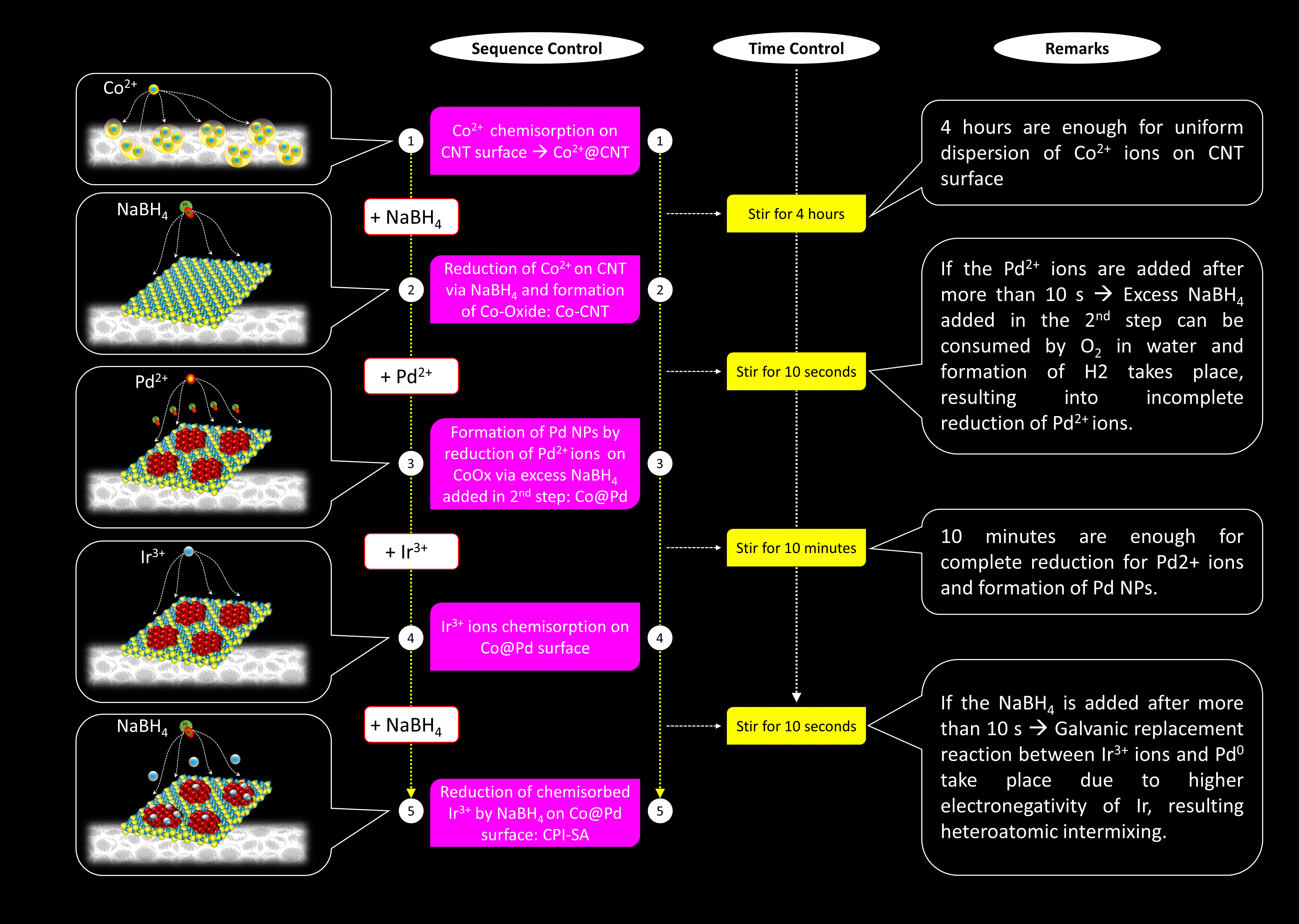
**

**Scheme S1.** The schematic representation of the synthesis process for CPI-SA catalyst.

1. **Physical Characterizations**

The atomic compositions of the CPI catalysts were investigated by an inductively coupled plasma-atomic emission spectrometer via ICP-AES (Jarrell-Ash, ICAP 9000). **The physical structures were determined by cross-referencing results of microscopy and X-ray spectroscopy techniques.** Aberration-corrected STEM characterization was performed on a Thermo-Fisher Themis Z microscope equipped with two aberration correctors under 300 kV. High angle annular dark-field (HAADF)-STEM images were recorded using a convergence semi angle of 11 mrad, and inner- and outer collection angles of 59 and 200 mrad, respectively. Energy-dispersive X-ray spectroscopy (EDS) was carried out using 4 in-column Super-X detectors. X-ray powder diffraction (XRPD) with synchrotron X-ray sources is employed for determining the crystal structure and corresponding patterns were collected at the beamline of BL-01C2 of National Synchrotron Radiation Research Center (NSRRC), Taiwan with the incident X-ray of wavelength 0.7749 Å (energy is 16 KeV). The X-ray absorption spectroscopy (XAS) was applied to unveil the electronic states and atomic arrangements. The typical XAS spectra of CPI catalysts at Ir L_3_-edge, Pd K-edge and Co K-edge were measured in fluorescence mode at beamlines BL-17C and 01C1 of NSRRC, Taiwan. X-ray photoelectron spectroscopy (XPS), carried out at beamline BL-24A1 of NSRRC, Taiwan, was used to investigate the oxidation states and surface compositions. **The proposed ORR pathways were** confirmed by cross-referencing the results of the *in-situ* partial fluorescence yield (PFY)-XAS inspections. The in-situ PFY-XAS inspection at Ir L_3_-edge, Pd K-edge and Co K-edge was performed at the beamline of BL-12XU with a customized single-compartment Teflon-made electrochemical cell at Spring-8, Japan. The samples for PFY-XAS analysis were prepared by dropping 300 micro-litre of catalyst slurry on the conducting graphite slit. The catalyst slurry was prepared with the same recipe as that of electrochemical measurements (*more details are given in the subsequent section*). During the measurement, the sample (i.e. catalyst-coated graphite plate) is placed in an electrochemical cell filled with oxygen-saturated electrolyte (0.1 M KOH). The sample side is faced with the incident X-ray where the window is sealed by a 10 μm thick Kapton tap.

1. **Electrochemical Analysis.**

An electrochemical cell (CH Instruments Model 600B, CHI 600B) equipped with a three-electrode system was used for all electrochemical measurements. The catalyst slurry for the ORR experiment was prepared by dispersing 5 mg of catalyst powder in 1.0 ml of isopropanol (IPA) containing 50 μl of Nafion-117 (99%, Sigma-Aldrich Co.) (as conducting binder). The as-prepared mixture was further subjected to ultrasonication for 30 minutes prior to the ORR test. For ORR measurements, 10.0 μl of catalyst slurry was drop cast and air-dried on a glassy carbon rotating disk electrode (RDE) (0.196 cm^2^ area) as the working electrode. Hg/HgCl_2_ (the voltage was calibrated by 0.242 V, in alignment with that of RHE) electrode saturated in KCl aqueous solution was used as a reference electrode. Whereas, a graphite rod was employed as the counter electrode to avoid any Pt contamination. The cyclic voltammetry (CV) and linear sweep voltammetry (LSV) were obtained at the voltage scan rate of 0.02 V s^−1^ and 0.001 V s^−1^, the potential range of 0.1 V to 1.3 V (V vs RHE.) and 0.4 V to 1.1 V (V vs RHE.), respectively, in an aqueous alkaline electrolyte solution of 0.1 M KOH (pH=13). The rotation rate of 400-3600 rpm was used for LSV. N_2_ and O_2_ atmosphere were used for CV and LSV, respectively. The durability of the experimental NCs was evaluated using an accelerated durability test (ADT) in the potential range of 0.5V to 1.0V (V vs RHE.) with the applied scan rate of 0.05 Vs^-1^ in O_2_ atmosphere. All the electrochemical experiments were performed at room temperature. The detailed procedure for the ORR mass activity calculation has been given in the following section.

For CO-stripping analysis, the adsorption of CO on the surface of the catalyst was performed initially by purging CO into 0.5 M H_2_SO_4_ at 0.05 V (vs RHE) for 20 min. Subsequently, the CO stripping voltammetry was measured between -0.10 and 1.20 V (vs RHE) in N_2_ saturated 0.5 M H_2_SO_4_ solution at a scan rate of 50 mVs^-1^. A glassy carbon electrode, Pt wire and Ag/AgCl electrode were used as the working, counter and reference electrodes, respectively.

1. **STEM images of Pd-CNT, Co@Pd and CPI-SA.**

For confirming the atomic distribution of Ir, Pd and Co, the STEM image and corresponding EDS map with the x-y line profiles and EDS maps are conducted by using high-resolution electron microscopy (Talos™ F200X G2 TEM in NSYSU). Accordingly, the Ir single atoms are uniformly dispersed both on the Co oxide support and the Pd NPs.


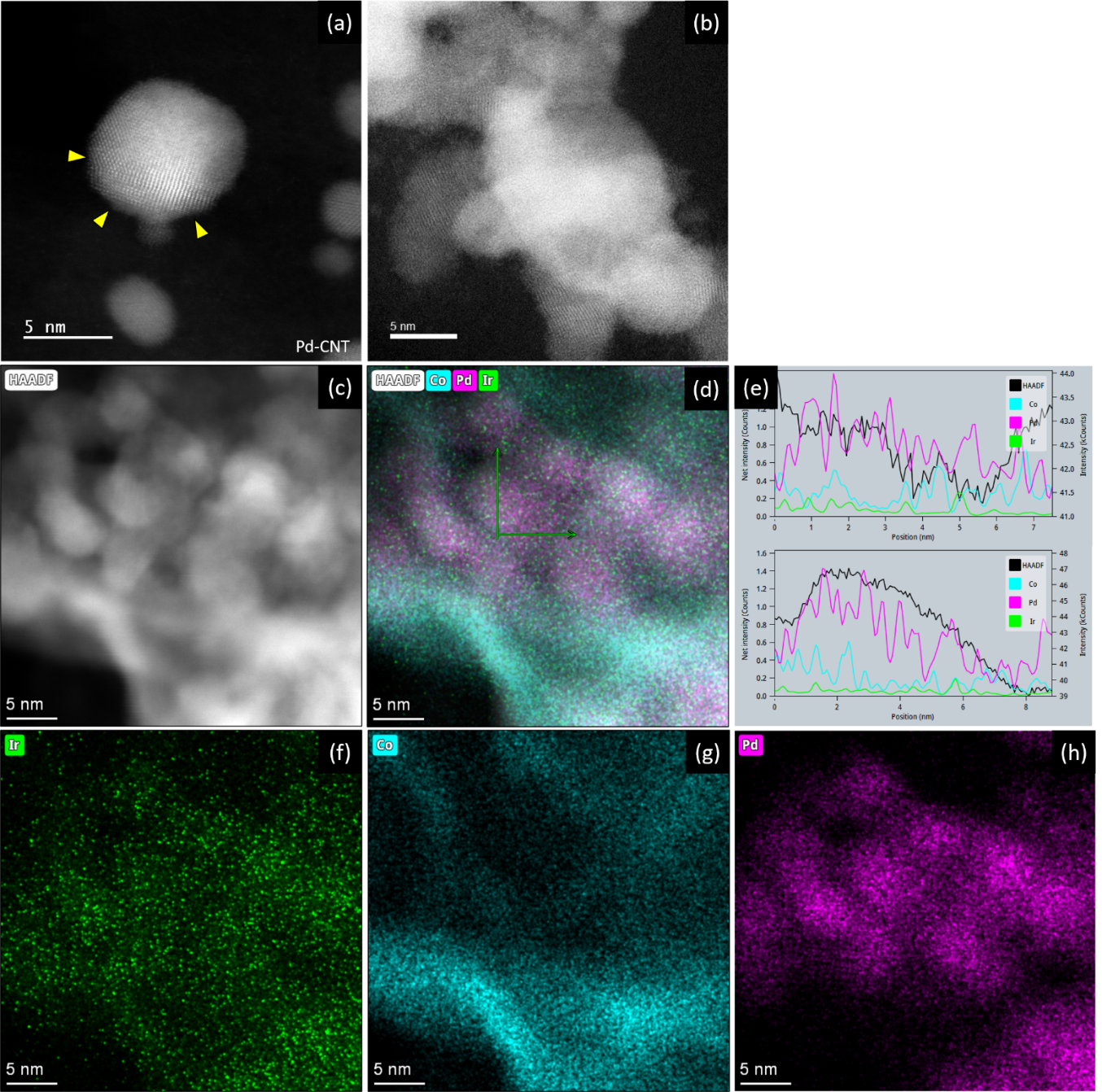


**Figure S1**. The aberrative-corrected high-angle annular dark-field scanning transmission electron microscopy HAADF-STEM images (a) Pd-CNT and (b) Co@Pd. (c) The STEM, (d) overlay for the EDS maps, (e) x-y line profiles across the Pd domain, and (f - h) the EDS maps of Ir, Co and Pd for CPI-SA.

1. **XRD Analysis.**


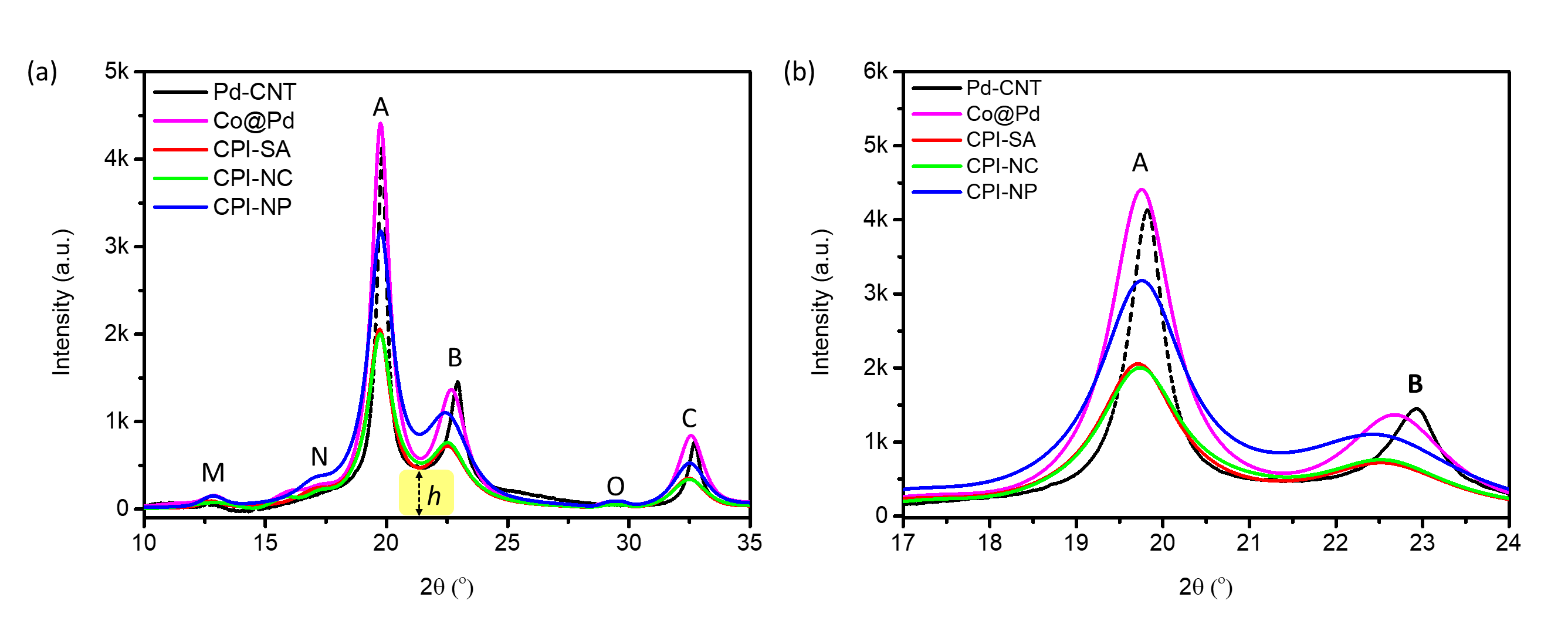


**Figure S2** (a) The comparative XRD patterns of CPI catalysts and reference samples (Pd-CNT and Co@Pd). The enlarged XRD spectra at different 2 theta range is presented in (b). The wavelength of the incident X-ray for data measurement is 0.7749 Å. The characteristic diffraction peaks M, N, A, B, O and C, respectively, correspond to contributions of the CNT (0001), Co_3_O_4_ (211), Pd (111), Pd (200), Co_2_Pd (1 0 -1 3) and the Pd (220) planes.

1. **XPS Analysis.**

Results of XPS analysis reveal the electronic structure of CPI catalysts as affected by decorated Ir-species with different dimensions. In this study, the excitation energy of the incident X-ray is 1000 eV corresponding to a probing depth of ~1.5 nm. The comparative XPS spectra of Co@Pd and CPI catalysts at Ir-4f, Pd-3d and Co-2p core levels are presented in **Figure S3**. In the Ir-4f spectrum (**Figure S3e**, **S3h** and **S3k**), the doublet peaks centred nearly around 61 eV and 63 eV are responses for photoelectron emission from Ir-4f_7/2_ and Ir-4f_5/2_ orbitals, respectively. These peaks are further deconvoluted to distinguish the photoelectron emission signals from different oxidation states of Ir and corresponding results are listed in **Table S1**. Accordingly, the CPI-SA preserves the highest index of Ir^3+^ species (Ir(III)) together with some metallic characteristics (i.e. Ir (0)). Besides, the metallic characteristics in CPI-NC and CPI-NP catalysts increase with the Ir-content increases from 1.0 to 7.0 wt.%, which is consistent with former physical inspection results. Meanwhile, as depicted in **Figures S3d**, **S3g** and **S3j,** no obvious change in the composition and binding energies of Pd are observed. Besides, it’s worth noticing that the signals of metallic Co (i.e. Co (0)) are enhanced (**Figure S3a**, **S3c**, **S3f** and **S3i**) with increasing dosage of Ir from 0 to 7 wt.%, revealing the shielding effect of Ir. Moreover, as shown in **Table S2**, the lowest binding energies of Ir^0^ and Ir ^3+^ strongly indicate the highest electron density of Ir in CPI-SA, which is expected to weaken the adsorption energy of intermediate species during ORR compared to CPI-NC and CPI-NP catalysts and thereby improve the ORR kinetics via facilitating the fast desorption of intermediates.


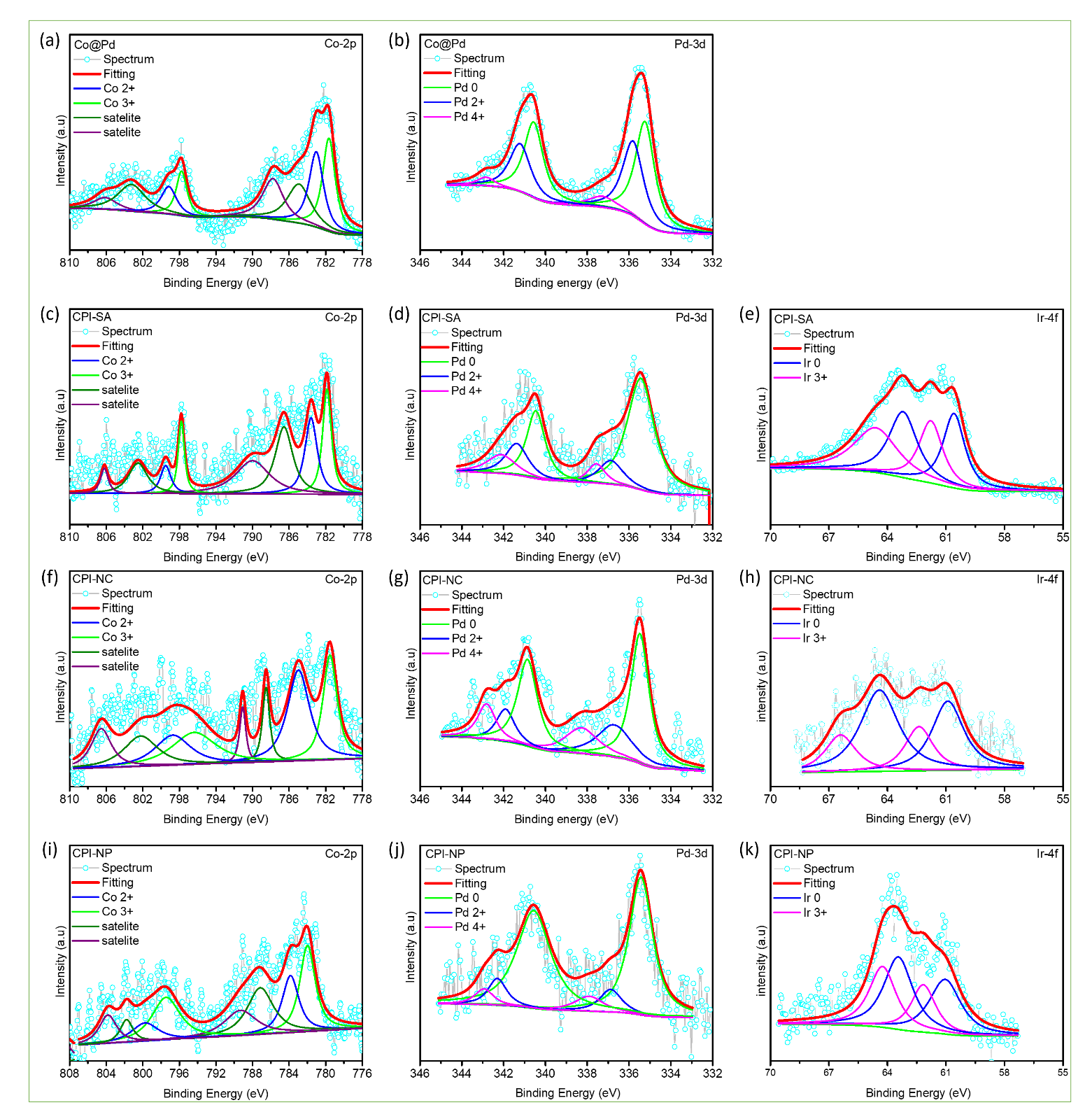


**Figure S3.** Comparative X-ray photoelectron spectroscopy of Co@Pd and CPI catalysts. (a) Co-2p orbital and (b) Pd-3d orbital of Co@Pd. (c) Co-2p orbital, (d) Pd-3d orbital and (e) Ir-4f orbital of CPI-SA. (f) Co-2p orbital, (g) Pd-3d orbital and (h) Ir-4f orbital of CPI-NC. (i) Co-2p orbital, (j) Pd-3d orbital and (k) Ir-4f orbital of CPI-NP.

**Table S1.** Comparative XPS determined composition ratios of Co@Pd and CPI catalysts.

| Samples |  | Surface Oxide Ratios (%) | | | | | | |  | Surface composition (%) | | |
| --- | --- | --- | --- | --- | --- | --- | --- | --- | --- | --- | --- | --- |
|  | Co (0) | Co (II) | Co (III) | Pd (0) | Pd (II) | Pd (IV) | Ir (0) | Ir (III) |  | Co | Pd | Ir |
| Co@Pd |  | 33.50 | 66.50 | 76.22 | 14.27 | 9.50 | N/A | |  | 61.59 | 38.41 | N/A |
| CPI-SA |  | 33.06 | 66.94 | 74.74 | 17.65 | 7.61 | 50.73 | 49.27 |  | 75.57 | 14.85 | 9.57 |
| CPI-NC | 7.85 | 26.81 | 65.34 | 66.75 | 22.30 | 10.95 | 61.39 | 38.61 |  | 79.48 | 13.00 | 7.51 |
| CPI-NP | 10.07 | 32.08 | 57.85 | 89.30 | 4.23 | 6.47 | 62.92 | 37.08 |  | 78.93 | 14.66 | 6.41 |

**Table S2.** Comparative XPS determined binding energies of Co@Pd and CPI catalysts.

| Samples |  | Binding Energy (eV) | | | | | | |  |
| --- | --- | --- | --- | --- | --- | --- | --- | --- | --- |
|  | Co (0) | Co (II) | Co (III) | Pd (0) | Pd (II) | Pd (IV) | Ir (0) | Ir (III) |  |
| Co@Pd | N/A | 782.75 | 781.34 | 335.39 | 336.58 | 337.85 | N/A | |  |
| CPI-SA | N/A | 782.90 | 781.38 | 335.52 | 336.71 | 337.98 | 60.47 | 61.49 |  |
| CPI-NC | 779.28 | 783.01 | 781.68 | 335.51 | 336.77 | 338.33 | 61.03 | 62.01 |  |
| CPI-NP | 779.27 | 782.98 | 781.67 | 335.56 | 337.17 | 338.16 | 60.72 | 61.54 |  |

1. **The Inductively coupled plasma-atomic emission spectrometer (ICP-AES) results.**

**Table S3**. Inductively coupled plasma-atomic emission spectrometer (ICP-AES) determined chemical composition of CPI catalysts.

| Samples | Co (wt.%) | Pd (wt.%) | Ir (wt.%) |
| --- | --- | --- | --- |
| CPI-1 | 11.72 | 29.59 | 0.887 |
| CPI-3 | 13.11 | 27.65 | 3.03 |
| CPI-7 | 12.98 | 26.15 | 6.855 |

1. **Model analysis fitting curves compared with experimental FT-EXAFS spectra at Ir L_3_-edge.**


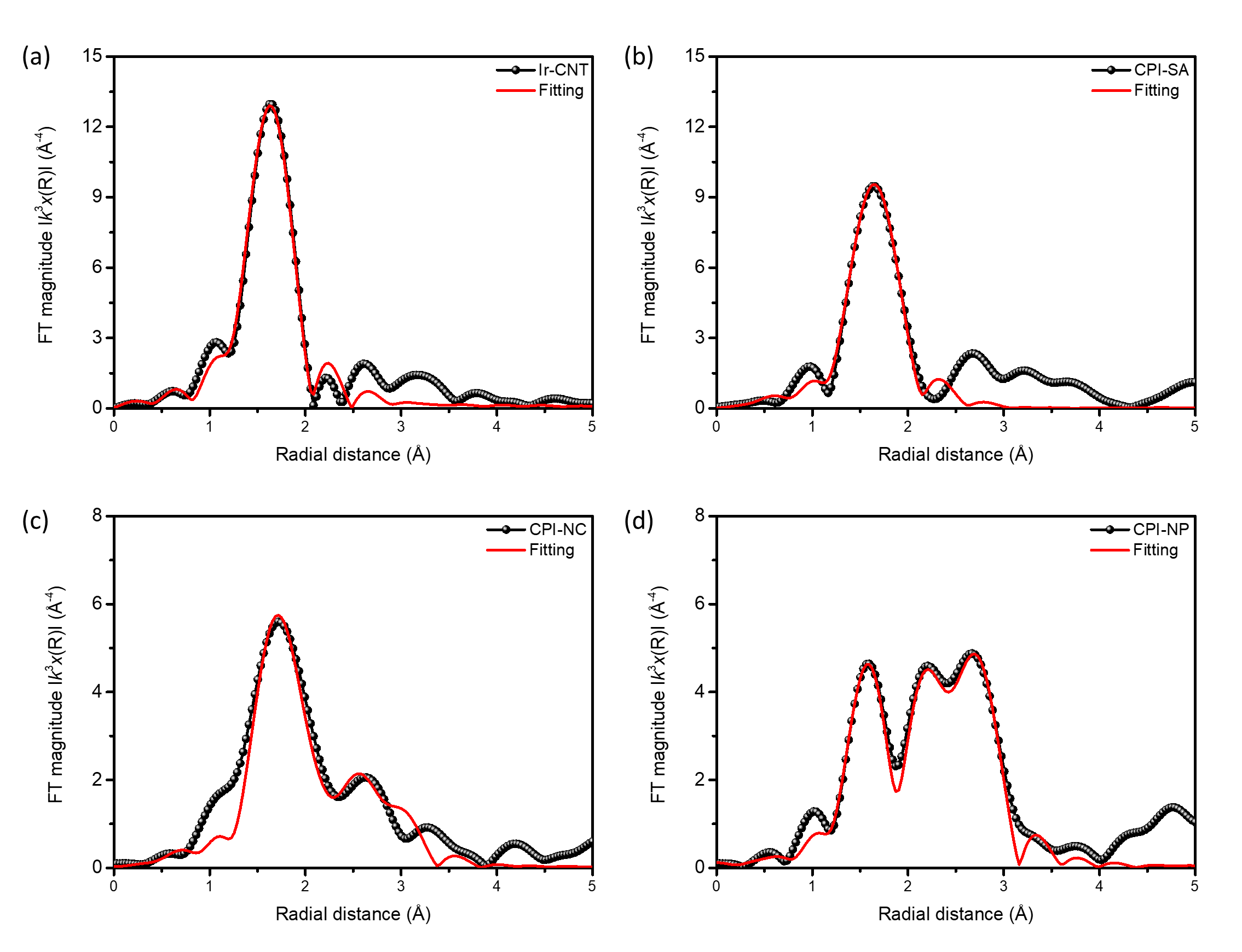


**Figure S4**. Model analysis fitting curves compared with experimental FT-EXAFS spectra at Ir L_3_-edge of (a) Ir-CNT, (b) CPI-SA, (c) CPI-NC and (d) CPI-NP catalysts.

1. **Model analysis fitting curves compared with experimental FT-EXAFS spectra at Pd K-edge.**


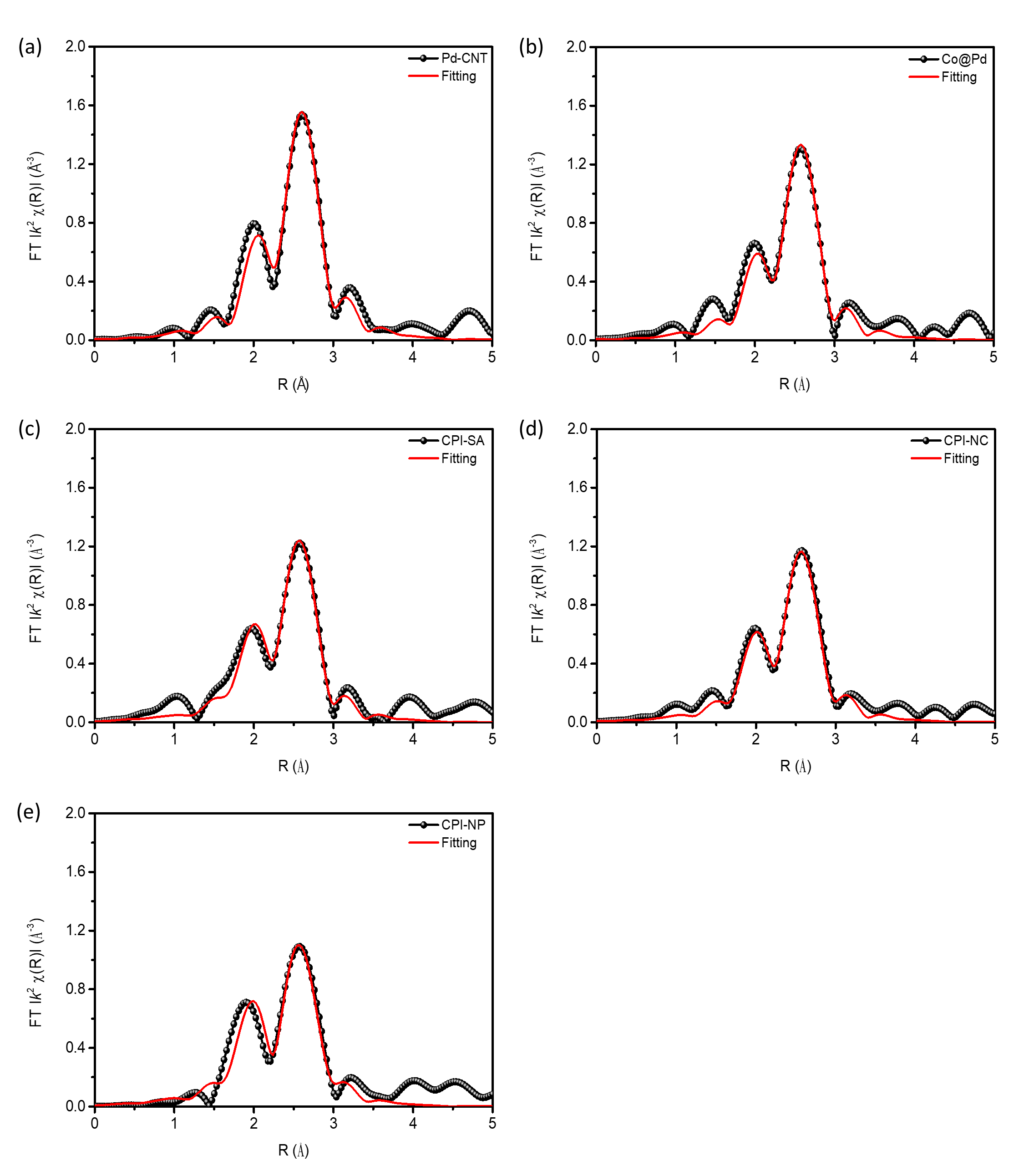


**Figure S5**. Model analysis fitting curves compared with experimental FT-EXAFS spectra at Pd K-edge of (a) Pd-CNT, (b) Co@Pd, (c) CPI-SA, (d) CPI-NC and (e) CPI-NP.

1. **Model analysis fitting curves compared with experimental FT-EXAFS spectra at Co K-edge.**


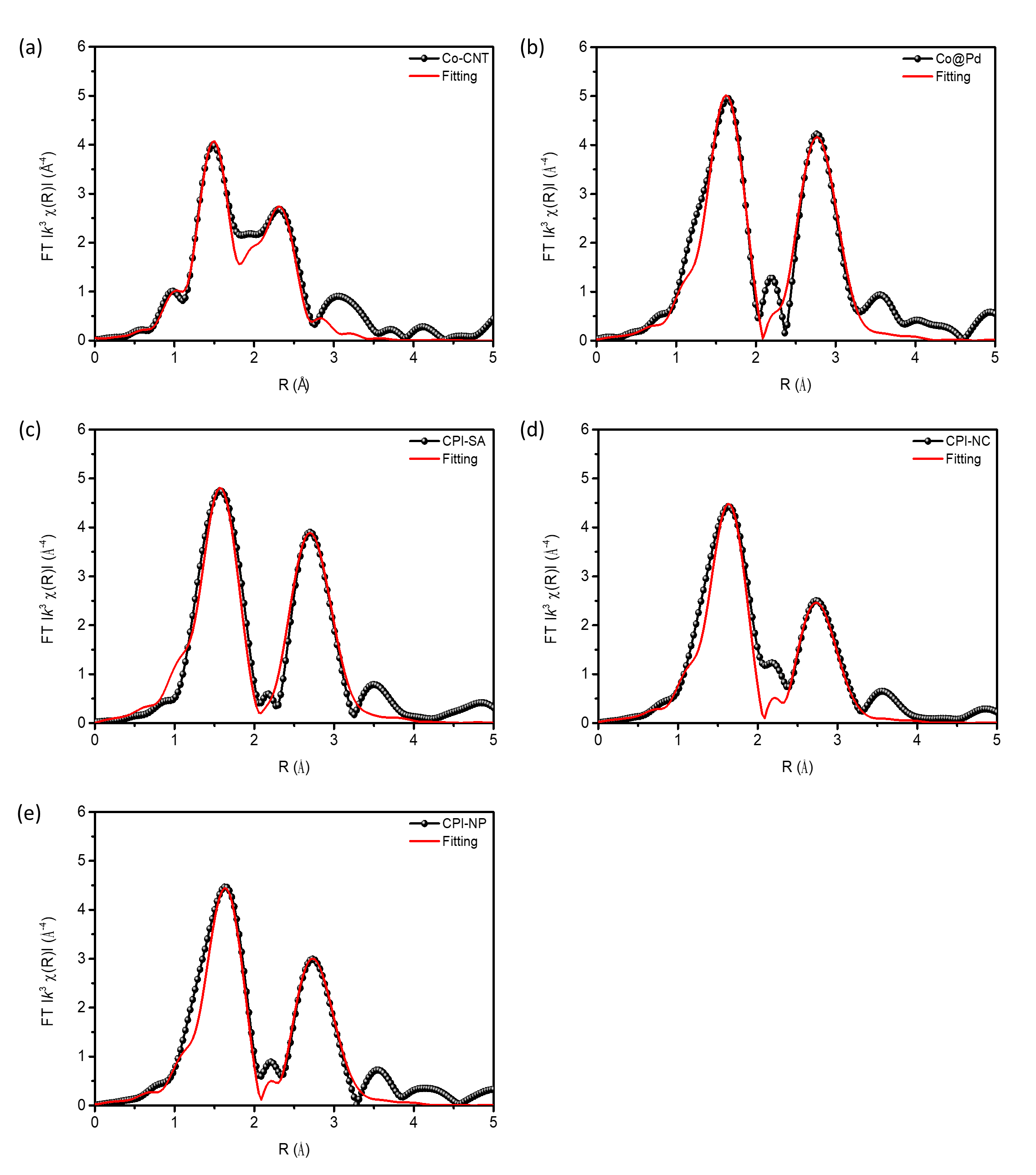


**Figure S6**. Model analysis fitting curves compared with experimental FT-EXAFS spectra at Co K-edge of (a) Co-CNT, (b) Co@Pd, (c) CPI-SA, (d) CPI-NC and (e) CPI-NP.

1. **Electrochemical results of reference samples.**


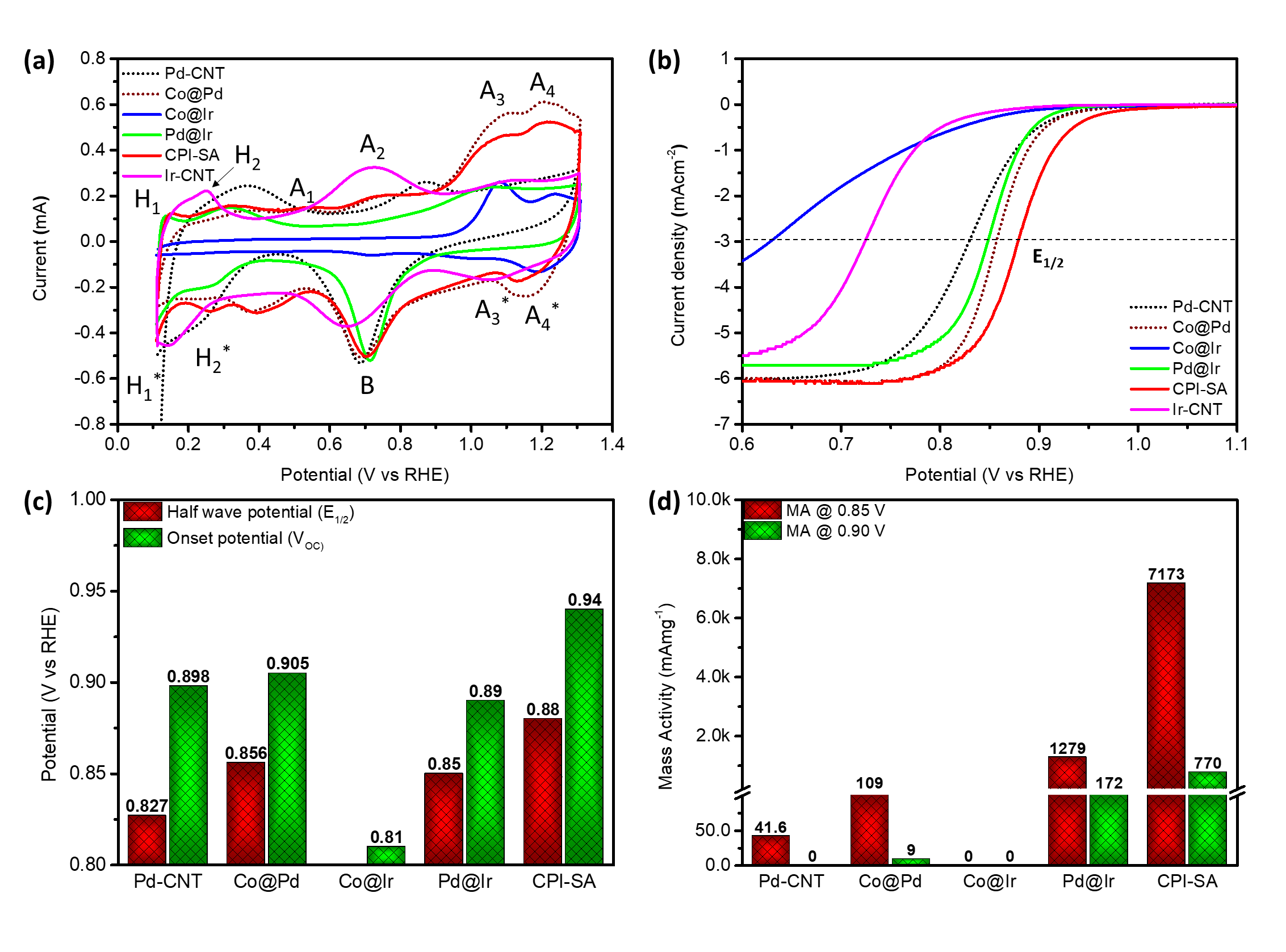


**Figure S7**. Electrochemical results of reference samples compared with CPI-SA. (a) CV, (b) LSV curves, (c) corresponding half-wave potential and onset potentials and (d) mass activities at 0.85 V and 0.9 V vs RHE.

**Table S4**. The electrochemical parameters of CPI catalysts and reference samples

| Sample* | V_oc 1600_ | E_1/2_ | J_k_ _(0.85, r)_ | J_k_ _(0.85, a)_ | MA_Pt (0.85)_ | MA_Ir (0.85)_ | MA_Pd (0.85)_ | MA_Pd+Ir (0.85)_ | J_k (0.90, r)_ | J_k (0.90, a)_ | MA_Pt (0.90)_ | MA_Ir (0.90)_ | MA_Pd (0.90)_ | MA_Pd+Ir (0.90)_ |
| --- | --- | --- | --- | --- | --- | --- | --- | --- | --- | --- | --- | --- | --- | --- |
|  | rpm(v) | _(V)_ | mAcm^-2^ | mAcm^-2^ | (mAmg^-1^) | (mAmg^-1^) | (mAmg^-1^) | (mAmg^-1^) | mAcm^-2^ | mAcm^-2^ |  | (mAmg^-1^) | (mAmg^-1^) | (mAmg^-1^) |
| J.M.-Pt/C | 0.910 | 0.844 | 4.37 | 4.37 | 67.0 |  |  |  | 1.17 | 1.17 | 24.9 |  |  |  |
| Co-CNT | N/A | N/A | N/A |  |  |  |  |  |  |  |  |  |  |  |
| Pd-CNT | 0.898 | 0.827 | 2.33 | 2.33 |  | N/A | 41.6 | N/A |  | 0 |  | N/A | 0 | N/A |
| Ir-CNT | 0.780 | 0.723 | 0.17 | 0.17 |  | 0 | N/A |  |  | 0 |  | 0 | N/A |  |
| Co@Pd | 0.905 | 0.856 | 5.15 | 5.15 |  | N/A | 109.0 |  | 0.66 | 0.66 |  | N/A | 9.3 |  |
| Co@Ir | 0.810 | 0.630 | 0.32 | 0.32 |  | 0 | N/A |  | 0.12 | 0.12 |  | 0 | N/A | N/A |
| Pd@Ir | 0.890 | 0.850 | 5.53 | 3.20 |  | 1279.0 | 57.8 | 55.3 | 0.43 | 0.43 |  | 171.5 | 7.8 | 7.4 |
| CPI-SA | 0.940 | 0.880 | 20.42 | 15.27 |  | 7173.4 | 215.9 | 209.6 | 2.30 | 1.64 |  | 770.4 | 23.2 | 22.5 |
| CPI-NC | 0.914 | 0.860 | 7.88 | 2.73 |  | 374.5 | 39.5 | 35.7 | 0.94 | 0.28 |  | 38.4 | 4 | 3.7 |
| CPI-NP | 0.908 | 0.846 | 4.56 | N/A |  |  |  |  | 0 | N/A |  |  |  |  |

* The parameters J_k_ _(0.85, r)_ and J_k_ _(0.85, a)_ correspond to the kinetics current of catalysts on the rotational electrode and from the active species at 0.85 V vs. RHE. For monometallic samples and Co@Ir (since Co oxide is chemically inactive in ORR), the two values are the same. For CPI catalysts, J_k_ _(0.85, a)_ is calculated by deducing the contribution of the J_k (0.85, r)_ of Co@Pd from that of CPI-i (where i stands for SA, NC and NP). A numerical representation for CPI-SA is J_k_ _(0.85, a)_ (CPI-SA) = J_k_ _(0.85, r)_ (CPI-SA) - J_k_ _(0.85, r)_ (Co@Pd) = 20.42 – 5.15 = 15.27 mAcm^-2^. Given that Pd@Ir is prepared by decorating Ir clusters in Pd-CNT, the J_k (0.85V, a)_ of (Pd@Ir) is determined as 3.20 mAcm^-2^ by the equation of J_k_ _(0.85, a)_ (Pd@Ir) = J_k_ _(0.85, r)_ (Pd@Ir) - J_k_ _(0.85, r)_ (Pd-CNT).

* The definition of J_k (0.90, r)_ and J_k (0.90, a)_ are the same to that of J_k (0.90, r)_ and J_k (0.90, a)_ excepting that the applied potential is 0.90 V vs. RHE.

1. **Calculation procedures for ORR mass activity**

It can be found that the Co-CNT does not show ORR activity at 0.85 V vs. RHE, whereas, J_k_ of the Co@Pd is 5.15 mA/cm^2^ (**Table S4**). Thus, considering the similar Pd content in the CPI catalysts as that of Co@Pd, we deducted the J_k_ of Co@Pd from the original J_k_ of CPI catalysts. In this way it is reasonable to conclude that the mass activities values summarized in **Table S4** and **Figure 4d** are comes from the decorated Ir species. For easy clarification, the numerical calculation procedures for the MA of CPI-SA has been presented below; where the original J_k_ at 0.85 V vs RHE is 20.42 mAcm^-2^ for CPI-SA (J_k_(CPI-SA, raw)) and 5.15 mAcm^-2^ for Co@Pd. Hence,

J_k_(CPI-SA) = J_k_(CPI-SA, raw) – J_k_(Co@Pd) = 20.42 - 5.15 = 15.27 mAcm^-2^

Now we used 15.27 as J_k_ for the calculation of MA for CPI-SA to ensure that MA_Ir_ is completely dominated by the decorated Ir single atoms.

The mass activity (MA) is calculated by following equation

$mass activity (mA mg^{-1})=J_{k}\times\frac{area}{mass of catalyst}$ (S1)

where *J*_k_ is the kinetic current density (mA/cm^2^) and area is the geometric area of working electrode (0.196 cm^2^). The mass activity of the catalyst is estimated via the calculation of *J*_k_ and normalization to the catalyst loading on glassy carbon rotating disk electrode.

With a significant difference between the kinetic current, the obtained MA for CPI catalysts is meaningful when Ir contents are less than 3.0 wt%. With the Ir content of 7.0 wt%, the CPI-NP exhibits J_k_ less than that of Co@Pd, which can be attributed to the strong affinity of metallic IrPd alloy and the Ir oxide (which are covered in the Co@Pd surface) to the chemisorbed O atoms and their shielding effects in the CPI-NP.

- Similarly, the mass activity of Pd@Ir is calculated after the deducing the J_k_ of Pd-CNT. In detail:

The original J_k_ of Pd@Ir at 0.85 V vs RHE = 5.53

The J_k_ of Pd-CNT at 0.85 V vs RHE = 2.33

Hence, J_k (Pd@Ir)_ = J_k_ _(Pd@Ir)_-J_k_ _(Pd-CNT)_ = 5.53-2.33 = 3.20

Now we used 3.20 as J_k_ for the calculation of mass activity for Pd@Ir NC to ensure that MA_Ir_ is completely coming from decorated Ir-clusters.

1. **The first derivative spectra of CPI catalysts.**


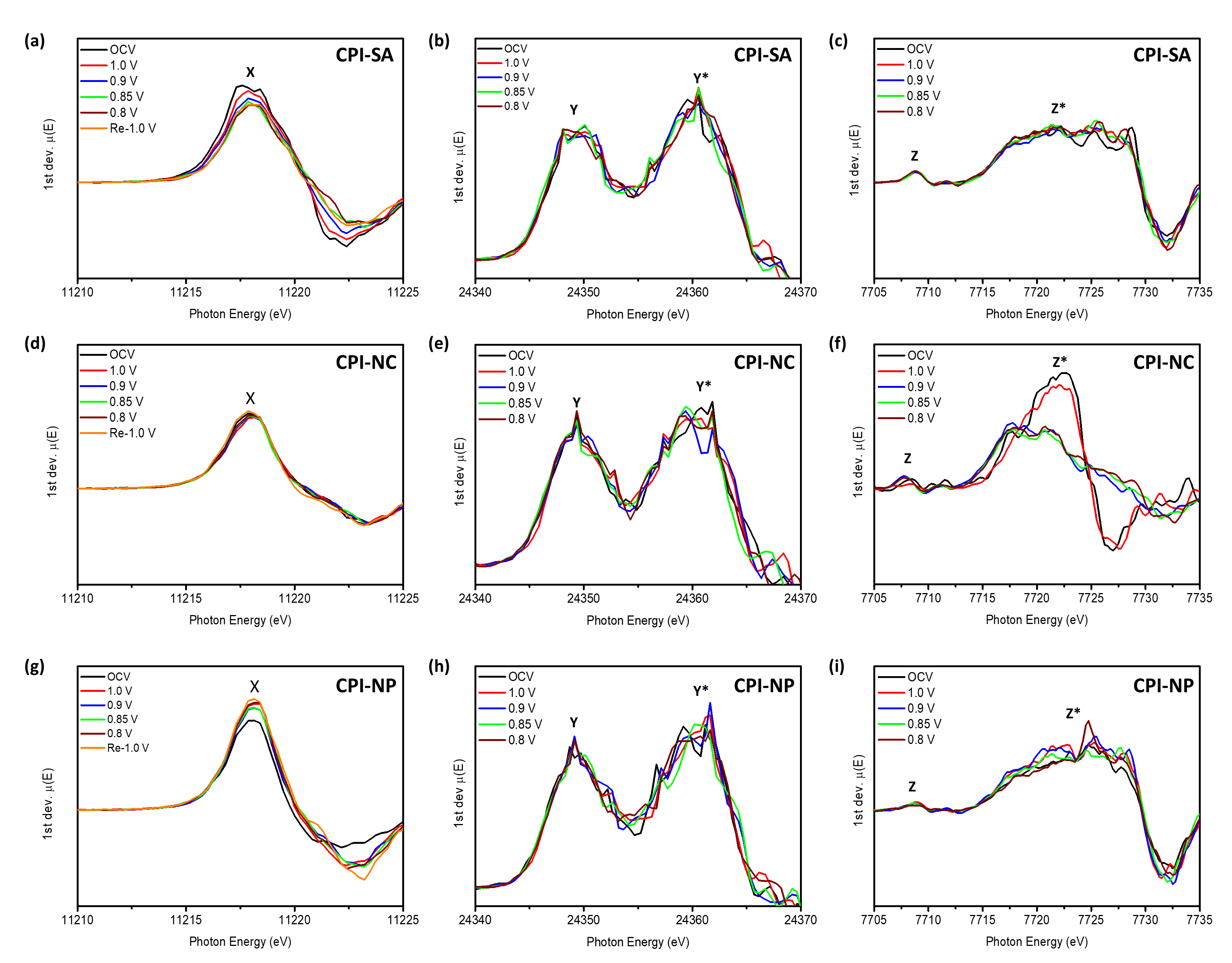


**Figure S8.** The first derivative spectra of CPI-SA at (a) Ir L_3_-edge, (b) Pd K-edge and (c) Co K-edge. The first derivative spectra of CPI-NC at (d) Ir L_3_-edge, (e) Pd K-edge and (f) Co K-edge. The first derivative spectra of CPI-NP at (g) Ir L_3_-edge, (h) Pd K-edge and (i) Co K-edge.

1. **In-situ PFY-XANES spectra of Co@Pd.**


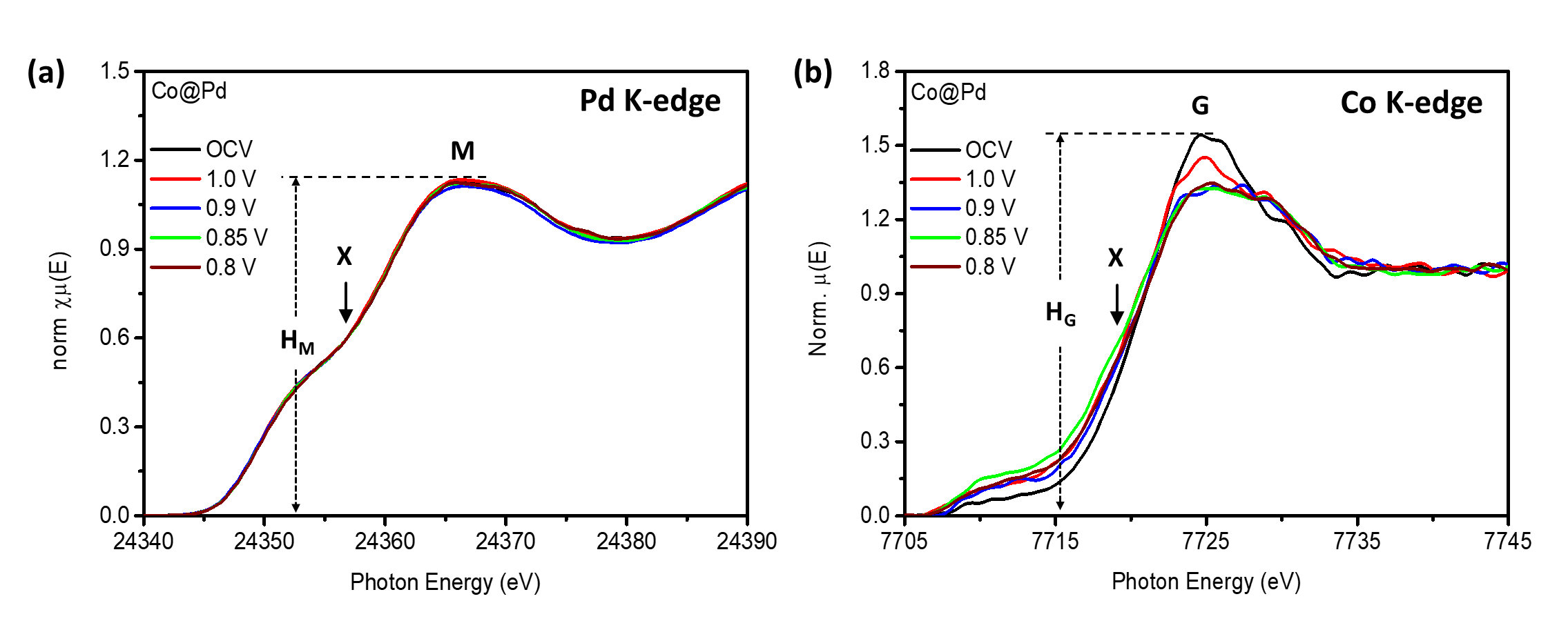


**Figure S9.** In-situ PFY-XANES spectra of Co@Pd at (a) Pd K-edge and (b) Co K-edge.

1. **In-situ PFY-XANES spectra of Ir-CNT and IrO_2_ at Ir L_3_-edge.**


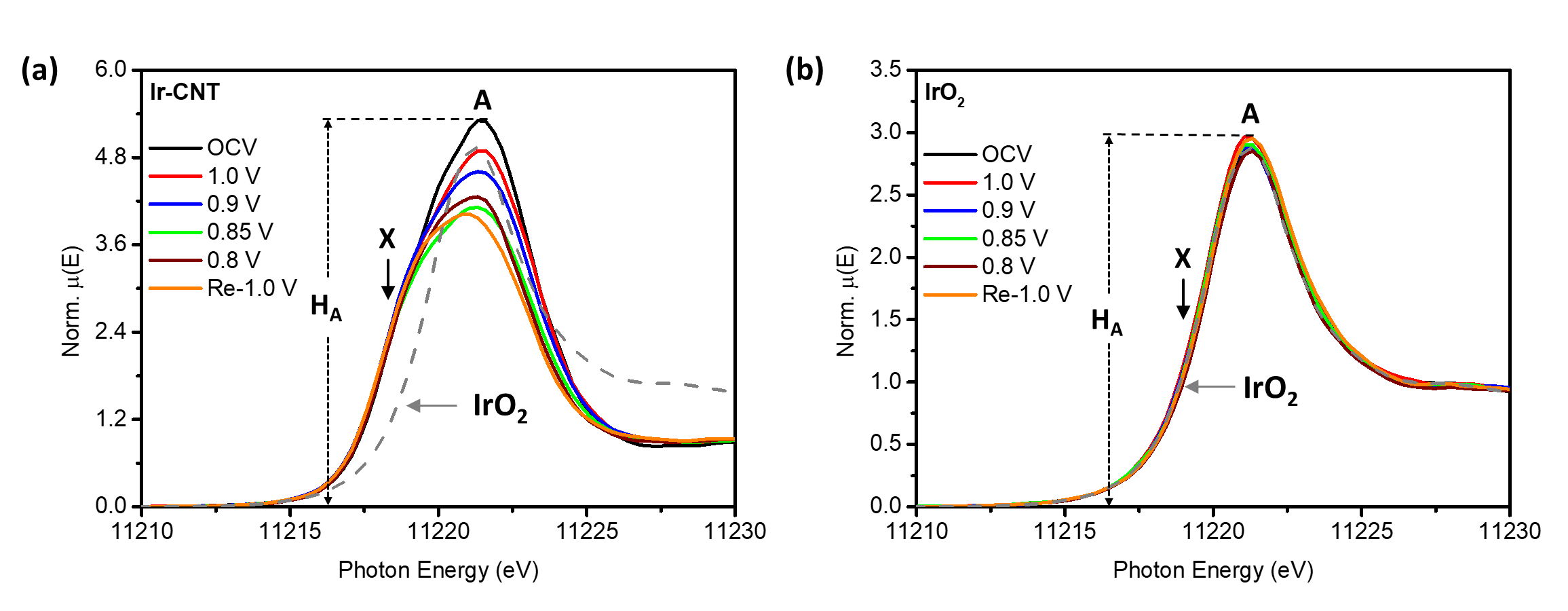


**Figure S10.** In-situ PFY-XANES spectra of (a) Ir-CNT and (b) IrO_2_ at Ir L_3_-edge.

1. **Comparison of selected catalysts in ORR.**

**Table S5. Comparison of ORR performance between CPI-SA and other catalysts in 0.1 M KOH electrolyte from literature.**

| **Catalysts** | **E_1/2_ vs RHE** | **V_OC_ vs RHE** | **MA (mAmg_Ir/Pt_^-1^)** | | **ADT Cycles** | **Stability** | **References** |
| --- | --- | --- | --- | --- | --- | --- | --- |
|  |  |  | **@ 0.85 V vs RHE** | **@ 0.90 V vs RHE** |  |  |  |
| CPI-SA | 0.883 | 0.94 | 7173 | 770 | 69 K | 100% | This Study |
| Ni@Pd-Ir | 0.861 | 0.91 | 2066 | N/A | 21 K | 95% | 1 |
| NPP-70 | N/A | | 1009.2 | 484.8 | 10 K | ∼ 100% | 2 |
| Co@Pd/Pt/CNT | 0.88 | 0.944 | 2055.1 | N/A | 310 K | 100% | 3 |
| H-Pt/CaMnO_3_ | 0.81 | 0.95 | 380 |  | 6 K | 95 % | 4 |
| Co@Pt | N/A | | 71.9 |  | 5 K | 17 mV penalty | 5 |
| Pd@PtNi/MWCNT |  |  | 73.3 |  | 5 K | 100 % | 6 |
| Pt-decorated three dimensional N-doped carbon |  |  | N/A | 162.88 | 5 K | 100 % | 7 |
| Pt@Pd nanocubes | 0.88 | 0.97 | 380 | N/A | 1 K | 80 % | 8 |
| Cu@Pd/Pt |  | 0.909 | 414 | N/A | | | 9 |
| PdCu_2_@Pt-H |  | N/A | 485 |  |  |  | 10 |

**References**

[1] D. Bhalothia, D.-L. Tsai, S.-P. Wang, C. Yan, T.-S. Chan, K.-W. Wang, T.-Y. Chen, P.-C. Chen, Ir-oxide mediated surface restructure and corresponding impacts on durability of bimetallic NiOx@Pd nanocatalysts in oxygen reduction reaction, Journal of Alloys and Compounds 844 (2020) 156160. https://doi.org/https://doi.org/10.1016/j.jallcom.2020.156160.

[2] D. Bhalothia, C. Yan, N. Hiraoka, H. Ishii, Y.-F. Liao, P.-C. Chen, K.-W. Wang, J.-P. Chou, S. Dai, T.-Y. Chen, Pt-Mediated Interface Engineering Boosts the Oxygen Reduction Reaction Performance of Ni Hydroxide-Supported Pd Nanoparticles, ACS Applied Materials & Interfaces 15(12) (2023) 16177-16188. https://doi.org/10.1021/acsami.2c21814.

[3] S. Dai, J.-P. Chou, K.-W. Wang, Y.-Y. Hsu, A. Hu, X. Pan, T.-Y. Chen, Platinum-trimer decorated cobalt-palladium core-shell nanocatalyst with promising performance for oxygen reduction reaction, Nature Communications 10(1) (2019) 440. https://doi.org/10.1038/s41467-019-08323-w.

[4] X. Han, F. Cheng, T. Zhang, J. Yang, Y. Hu, J. Chen, Hydrogenated Uniform Pt Clusters Supported on Porous CaMnO3 as a Bifunctional Electrocatalyst for Enhanced Oxygen Reduction and Evolution, Advanced Materials 26(13) (2014) 2047-2051. https://doi.org/https://doi.org/10.1002/adma.201304867.

[5] L. Wang, Z. Tang, W. Yan, Q. Wang, H. Yang, S. Chen, Co@Pt Core@Shell nanoparticles encapsulated in porous carbon derived from zeolitic imidazolate framework 67 for oxygen electroreduction in alkaline media, Journal of Power Sources 343 (2017) 458-466. https://doi.org/https://doi.org/10.1016/j.jpowsour.2017.01.081.

[6] S. Liu, Y. Wang, L. Liu, M. Li, W. Lv, X. Zhao, Z. Qin, P. Zhu, G. Wang, Z. Long, F. Huang, One-pot synthesis of Pd@PtNi core-shell nanoflowers supported on the multi-walled carbon nanotubes with boosting activity toward oxygen reduction in alkaline electrolyte, Journal of Power Sources 365 (2017) 26-33. https://doi.org/https://doi.org/10.1016/j.jpowsour.2017.08.073.

[7] Y. Cheng, H. Lu, K. Zhang, F. Yang, W. Dai, C. Liu, H. Dong, X. Zhang, Fabricating Pt-decorated three dimensional N-doped carbon porous microspherical cavity catalyst for advanced oxygen reduction reaction, Carbon 128 (2018) 38-45. https://doi.org/https://doi.org/10.1016/j.carbon.2017.10.102.

[8] C.-L. Lee, C.-C. Yang, C.-R. Liu, Z.-T. Liu, J.-S. Ye, Pt-coated Pd nanocubes as catalysts for alkaline oxygen reduction activity, Journal of Power Sources 268 (2014) 712-717. https://doi.org/https://doi.org/10.1016/j.jpowsour.2014.06.112.

[9] H.-Y.T. Chen, J.-P. Chou, C.-Y. Lin, C.-W. Hu, Y.-T. Yang, T.-Y. Chen, Heterogeneous Cu–Pd binary interface boosts stability and mass activity of atomic Pt clusters in the oxygen reduction reaction, Nanoscale 9(21) (2017) 7207-7216. https://doi.org/10.1039/C7NR01224A.

[10] H.-Y. Park, J.H. Park, P. Kim, S.J. Yoo, Hollow PdCu2@Pt core@shell nanoparticles with ordered intermetallic cores as efficient and durable oxygen reduction reaction electrocatalysts, Applied Catalysis B: Environmental 225 (2018) 84-90. https://doi.org/https://doi.org/10.1016/j.apcatb.2017.11.052.
